# The effect of robot-assisted gait training on physical activity outcomes in people with spinal cord injury: A systematic review

**DOI:** 10.1177/02692155251411864

**Published:** 2026-02-18

**Authors:** James Belsey, Andrew Reid, Scott Hannah, Louise Johnson, James Faulkner

**Affiliations:** 1 Department for Allied Health Professions, Social Work and Sport, 8629 University of Winchester, Winchester, UK; 2 School of Sciences, 1554 Bath Spa University, Bath, UK; 3 Stroke Services, University Hospitals Dorset NHS Foundation Trust, Bournemouth, UK; 4 Primary Care Research Centre, 7423 University of Southampton, Southampton, UK

**Keywords:** Spinal cord injury, robot-assisted gait training, gait, exercise training, functional outcome, exoskeleton, exertion, walk test

## Abstract

**Objective:**

To summarise the evidence for changes in physical activity outcomes during robot-assisted gait training in patients with spinal cord injury.

**Data sources:**

The Web of Science, Physiotherapy Evidence Database, Central, Medline, Scopus and SportDiscus databases were searched in August 2025 for studies that recorded ≥1 physical activity outcome during robot-assisted gait training.

**Review methods:**

Data were synthesised according to the Synthesis Without Meta-analysis guidelines. Risk of bias was assessed using the Physiotherapy Evidence Database scale or the Revised Risk of Bias Assessment Tool for Non-Randomised Studies. Certainty of evidence was established following the Grading of Recommendations, Assessment, Development and Evaluations framework. The report followed the Preferring Reporting Items for Systematic Reviews and Meta-Analyses guidelines.

**Results:**

Thirty studies (638 participants) were eligible for inclusion. Quality of the randomised studies ranged from ‘Fair’ to ‘Good’, while there was high risk of bias for all non-randomised studies in ≥1 domain. Robot-assisted gait training significantly improved physical activity outcomes (up time, walk time, walk distance, walk speed and number of steps) over time, though these findings were constrained by very low certainty of evidence.

**Conclusion:**

Up time, walk time, walk distance, walk speed, and number of steps were significantly improved across the robot-assisted gait training period for patients with spinal cord injury. Robot-assisted gait training during rehabilitation for people following spinal cord injury is a useful adjunct to support independence and improved walking ability.

## Introduction

Spinal cord injury is a serious neurological condition caused by damage to the vertebrae or surrounding tissue, which was recently estimated to have an incidence rate of 23.77 (95% confidence interval [95% CI]: 21.50–26.15) per 1,000,000 people globally.^1^ Depending on the level and severity of spinal cord injury, patients can be affected to different degrees ranging from irreversible disability to impaired mobility and function that may improve over time.^2,3^ Among other effects, spinal cord injury is often associated with a partial or total loss in walking ability and is, therefore, of primary concern within rehabilitation protocols.^4–6^ This reduced walking ability leads to a sedentary lifestyle, which is linked to various physical co-morbidities, a decline in quality of life, and an increase in healthcare costs.^3,7,8^ While quality of life is affected by a combination of mental and physical health, mental health has been shown to improve to a greater degree than physical health over time, which remains low and negatively affects health-related quality of life.^7^

A wide variety of assistive technologies are currently used to provide physical therapy to people with spinal cord injury, with their inclusion in gait training being common to help restore function and walking ability.^3^ Robot-assisted gait training, via the use of mechanical exoskeletons, is one such technology that has shown promise in comparison to conventional physiotherapy.^9–12^ Several systematic reviews and meta-analyses have demonstrated the effectiveness of robot-assisted gait training on functional^10,13–15^ and cardiovascular outcomes for patients with spinal cord injury.^16,17^ Functional outcomes like the 6-minute walk test and 10-metre walk test have sometimes been used as measures of walk distance and speed to summarise changes in walking ability before and after robot-assisted gait training.^18–21^ However, these functional tests do not provide insight into the actual physical activity engagement spinal cord injury participants are exposed to during robot-assisted gait training interventions.

Considering that reducing sedentary time is particularly relevant for the numerous health-related outcomes outlined above, it is important to examine the role of robot-assisted gait training in supporting spinal cord injury rehabilitation. In line with the ‘PICO’ criteria,^22^ the aim of this systematic review was therefore to summarise the evidence for patients with spinal cord injury who underwent robot-assisted gait training, for whom changes in physical activity outcomes were evaluated within training sessions at the beginning, during, or at the end of the training period.

## Method

This systematic review was prospectively registered on the PROSPERO registry (ID: CRD42023382402) and was conducted according to the 2020 version of the Preferred Reporting Items for Systematic Reviews and Meta-Analyses guidelines.^23,24^ The narrative reporting of the data synthesis methods followed the Synthesis Without Meta-Analysis for systematic reviews guidelines.^25^ Finally, the Grading of Recommendations, Assessment, Development and Evaluation framework was applied using the approach of Murad et al.^26^ to provide a rating of the certainty of evidence for the outcomes extracted from included studies.^27,28^

### Search strategy

All peer-reviewed publications, irrespective of study design or publication language, that involved adult (≥18 years of age) human participants with a clinically diagnosed spinal cord injury, who underwent some form of robot-assisted gait training as part of their rehabilitation, were considered potentially eligible for this systematic review. Previously published review articles or meta-analyses that appeared in the search results were not included in the final review. Studies involving a combination of neurological diseases, or people above and below 18 years of age, were only included if the data for the relevant participants could be separated.

To be included in the final review, at least one physical activity outcome during robot-assisted gait training needed to be reported. Physical activity outcomes were defined as any commonly used indicator of activity intensity or fitness such as: metabolic equivalents, walk distance, walk speed, number of steps and other gait parameters.^29,30^ Where outcomes were reported at multiple time points, only data from the beginning and end of the period were extracted so the effect of robot-assisted gait training over time could be examined. Physical activity outcomes that were recorded during functional tests (e.g., 6-minute walk test), or outside of the robot-assisted gait training period, were similarly not eligible for inclusion.

The Web of Science, MEDLINE, Cochrane Register of Controlled Trials, SportDiscus, Physiotherapy Evidence Database, and Scopus databases were initially searched in August 2023 with subsequent searches using identical terms and databases being conducted in June 2024 and August 2025 to ensure that any relevant articles published since the original search could also be included. A manual search through the reference lists of relevant review articles and meta-analyses from the results was also conducted.

**Figure 1.**
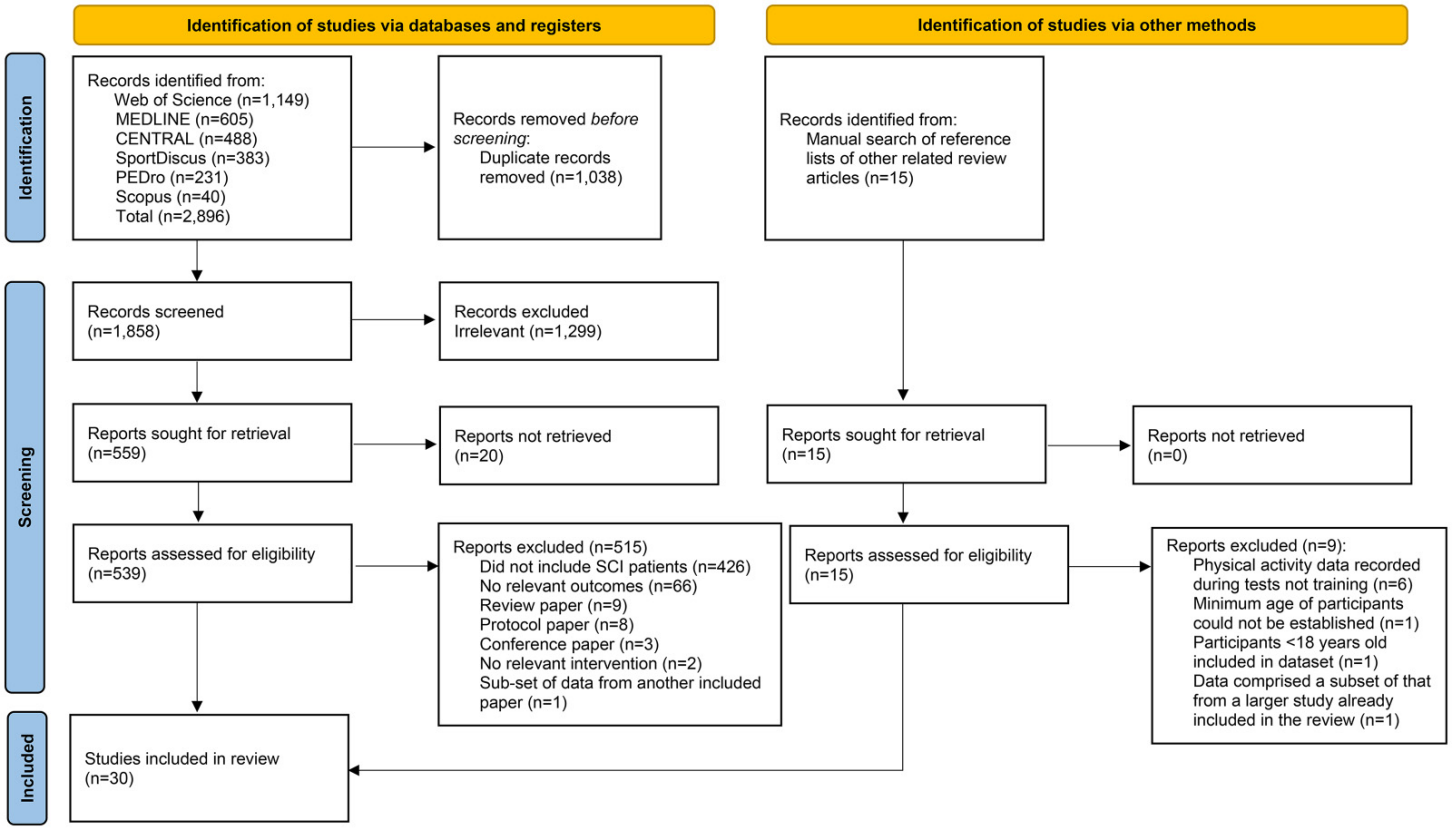
PRISMA flowchart for the literature screening process.^23^

With the exception of the Physiotherapy Evidence Database, the following search terms were entered into all of previously mentioned databases and registers: (brain injuries OR cerebral palsy OR multiple sclerosis OR Parkinson's disease OR spinal cord injury OR stroke OR cerebrovascular accident OR neurological) AND (robot* assisted gait train* OR robot-assisted gait training OR RGT OR electromechanic* assisted gait training OR EGT OR EMGT OR exoskeleton OR end effector) AND (physical activity OR exercise OR intensity OR activity level OR sedentary). Where possible, MeSH terms were used for the part of the search related to the neurological conditions. The searches were originally conducted as part of a wider literature search involving a range of neurological conditions for a PhD project (AR). Therefore, the strategy was broader than strictly necessary for the initial search results. For the purposes of this systematic review, articles that only involved people with neurological conditions other than SCI were excluded at the screening stage (Figure 1).

The Physiotherapy Evidence Database is more limited in its functionality than the other sources used for this review. Consequently, four separate searches were conducted using the following simpler terms to encapsulate all relevant literature: ‘robot assisted gait training’, ‘electromechanical gait training’, ‘exoskeleton’, and ‘end effector’.

### Article screening and data collection

The systematic review management software Covidence (Veritas Health Innovation, Australia) was used to store the search results and track the screening process. Two reviewers (AR and JF) independently screened the title and abstract of each record to eliminate any clearly irrelevant articles from the review, before conducting a final round of screening based on the full-text of the remaining articles. For this final round of screening, a third independent reviewer (JB) was also involved. Any differences in the number of articles excluded at each phase of screening by the reviewers were addressed by meeting to discuss the differences. If the differences still could not be resolved, an additional reviewer (SH or LJ) moderated the discussion until consensus was achieved.

Potentially relevant articles published in languages other than English or German were first translated into English using Google Translate before being screened and either included or excluded, as appropriate.

A single reviewer (JB) manually extracted the following data from each study and organised them using spreadsheet software (Microsoft Excel, Microsoft, USA): inclusion/exclusion criteria, methods, participants, intervention, outcomes, funding, and conflict of interest statements. A second reviewer (AR) independently collected the same data from a sample of the included articles to confirm that the original data collection process had been conducted appropriately and consistently.

Where articles matched the eligibility criteria for inclusion but did not present data in a way that allowed extraction, the authors of those studies were contacted via email to request the necessary information. In instances where data were presented in graphical form, the online artificial intelligence-assisted tool WebPlotDigitizer v5 (https://automeris.io/) was used as a valid and reliable method to extract numerical data.^31,32^

### Risk of bias assessment

The Physiotherapy Evidence Database scale was used to assess the risk of bias for included studies with a randomised design.^33,34^ For included studies with non-randomised study designs, the Revised Risk of Bias Assessment Tool for Nonrandomized Studies was applied as it is sensitive to different study designs.^35^ Results from the assessments with the latter tool were presented in graphical form using a specialist visualisation software.^36^ Finally, all included articles were evaluated in terms of their inherent level of evidence, in accordance with established evidence-based medicine guidelines.^37^

Two reviewers (JB and JF) conducted the risk of bias and level of evidence evaluations independently. Disagreements were initially addressed through discussion and resolved with an additional moderated round of discussion, where necessary.

### Data synthesis

Studies that measured ≥1 outcome of interest were grouped together per outcome to provide a summary of the observed change over time. An investigation of heterogeneity was not pre-specified for this review, though an informal assessment based on the extracted study characteristics was performed.

Data related to statistical analyses were extracted (e.g., *p* values, 95% CI), where reported. Where this was not possible, descriptive statistics (e.g., mean and standard deviation) were extracted to allow the overall direction of effect to be established.

Two reviewers (JB and JF) performed the certainty of evidence assessment independently. Disagreements were initially addressed through discussion and resolved with an additional moderated round of discussion, where necessary.

## Results

A total of 2896 publications were identified from the databases and manual searches before being screened for duplicates and eligibility (Figure 1). A full summary of the characteristics of the final 30 studies included in the review can be found in Table 1. Two of the three studies rated with the highest level of evidence were pilot studies,^38,39^ while the most common level of evidence was level IV (n = 15 studies; Table 1).

**Table 1.** Characteristics of the included studies (n = 30).

Authors (date)	Design (level of evidence)	Sample size (*n*)	Age (y)	Sex (M:F)	Level of injury	ASIA impairment scale	Time since injury (months)	RAGT device	RAGT session information	Physical activity outcomes	Functional walk tests	Time points
Aach et al. (2023)	Single group longitudinal feasibility study (IV)	50	43.9 ± 15.0	36:14	Cervical n = 17; Thoracic n = 24; Lumbar n = 9;	A, n = 3 C, n = 30 D, n = 17	3.9 ± 3.2	Hybrid Assistive Limb	60 ± 30 sessions of 90 min, 5 per week	Walk speed; walk time; distance	6MWT; 10MWT; TUG	First and last sessions
Bonnevie et al. (2025)	Single group observational pilot study (IV)	5	55 range 28-59	2:3	Not reported	Not reported	148.8 range 76-222	LEXO	One session Median 18 min (IQR 12-29)	Steps	None	Cumulative total within session
Bosteder et al. (2023)	Single intervention case series(IV)	2	24.5 ± 5.0	2:0	Cervical n = 2	A, n = 2	73 ± 100.4	Ekso GT	One1 session	Up time; walk time; steps; METs; METs during MVPA; duration of MVPA; % of session as MVPA	None	Average across session
Chang et al. (2020)	Case study (V)	1	66	1:0	Cervical	A	83	Ekso	Nine sessions of 60 min in a 3 week period	Up time; walk time; steps; step speed	None	Each session

Faulkner et al. (2021)	Parallel group pre-post pilot study (II)	12	34 ± 15	6:6	Not reported	Not reported	37.8 ± 22.8	Ekso	5 days (consecutive) RAGT group: 60 min physio + 90 min RAGT Control group: 60 min physio + 60 min home exercises	Up time; walk time; steps; level of motor assistance (RAGT group only)	None	First and last sessions
Fleerkotte et al. (2014)	Single group pilot study (IV)	12	47.1 ± 11.6	4:6	Cervical n = 3; Thoracic n = 4; Lumbar n = 3	B, n = 1 C, n = 5 D, n = 4	46.3 ± 42.2	Lower Extremity Powered Exo-skeleton (LOPES)	Mean 20 sessions (range 18–24) of up to 60 min each 3 per week for 8 weeks	Walk time; BW support; impedance level; walk speed; cycle time; step symmetry index; step width; step length; relative stance phase duration; maximum knee flexion during swing phase; knee RoM during stance phase; hip RoM	6MWT; 10MWT; TUG	First and last sessions

Gagnon et al. (2018)	Single group longitudinal feasibility study (IV)	14	38.7 ± 10.9	9:4	Cervical n = 1; Thoracic n = 13	A, n = 13 B, n = 1	88.8 ± 93.6	Ekso	17 ± 2 sessions of 60 min 3 per week for 6 weeks	Up time; walk time; steps	10MWT	Average across all sessions; mean during first and last two sessions
Gillespie et al. (2023)	Retrospective single group longitudinal study (IV)	99	44 ± 17.8	72:27	Cervical n = 55; Thoracic n = 34; Lumbar n = 5; Unknown n = 3	A, n = 12 B, n = 14 C, n = 23 D, n = 24 Unknown, n = 26	3.1 ± 7.2	Ekso GT or Ekso NR	Mean 6 ± 4 sessions of 45 min Mean 1 ± 1 sessions per week	Up time; walk time; steps	None	First, fifth, and last sessions
Grasmücke et al. (2017)	Longitudinal with sub-group analysis (IV)	70	44.3 ± 13.9	43:12	Cervical n = 11; Thoracic n = 22; Lumbar n = 20	A, n = 18 C, n = 24 D, n = 13	6.9 ± 5.1	Hybrid Assistive Limb	Mean 59 ± 2 sessions of 90 min 5 per week for 12 weeks	Walk speed; walk time; walk distance	6MWT; 10MWT	First, sixth, and last week
Hong et al. (2020)	RCT crossover (II)	50	38.7 ± 14.2	38:12	Not reported	A/B, n = 31 C/D, n = 19	56.3 ± 62.2	ReWalk and Ekso	36 sessions of 90–120 min 3 per week for 12 weeks	Steps	6MWT; 10MWT; TUG	Overall; first, 18th, and last sessions; sessions 1–12, sessions 13–24, and sessions 25–36

Hotz et al. (2024)	Mixed methods feasibility study (IV)	2	55.6 ± 9.9	1:1	Lumbar	D, n = 2	118.8 ± 142.8	LEXO	45 min per session 4 per week for 4 weeks	Walk distance; steps; step length; up time; walk speed	6MWT; 10MWT; TUG	First and last sessions
Ikumi et al. (2017)	Pre-post case study (V)	1	19	1:0	Cervical	A, n = 1	48	Hybrid Assistive Limb	10 sessions of 60 min 2 per week for 5 weeks	Walk time; walk distance	None	First and last sessions
Karelis et al. (2017)	Single group pre-post intervention (IV)	5	60.4 ± 6.1	4:1	C7-T10 n = 5	A, n = 5	91.2 ± 55.2	Ekso	60 min sessions 3 per week for 6 weeks	Up time; walk time; steps	None	Average across all sessions
Khan et al. (2019)	Single group longitudinal study (IV)	12	37.5 ± 13.7	8:4	Cervical n = 3; Thoracic n = 9	A, n = 6 B, n = 2 C, n = 3 D, n = 1	91.2 ± 97.2	ReWalk 2.0 and 5.0	52 ± 6 sessions over a 12 week period	Walk speed; walk distance; steps; steps/bout of walking without stopping	6MWT; 10MWT	First, 12th and last week
Kolakowsky-Hayner et al. (2013)	Single group longitudinal study (IV)	8	29.9 ± 6.9	5:2	Thoracic n = 7	A, n = 7	10.2 ± 7.2	Ekso	Six sessions; 1 per week 20 min in first session; 40 min in second session; 60 min in other sessions	Up time; walk time; walk speed; walk distance; step length	None	Each session
Kressler et al. (2014)	Longitudinal case series (IV)	3	30.3 ± 6.7	2:1	Thoracic n = 3	A, n = 3	Not reported	Ekso	18 sessions of 60 min 3 per week	Up time; walk time; walk distance; steps	2MWT; 10MWT	First and last sessions; average across all sessions
Lam et al. (2015)	Double blind RCT pilot (I)	17	44.8 ± 14.6	9:4	Cervical n = 10; Thoracic n = 5	C, n = 5 D, n = 10	64.8 ± 63.1	Lokomat	Mean 36 ± 1 sessions of 45 min 3 per week for 3 months	Treadmill speed; RPE	6MWT; 10MWT	First and last week
Lemaire et al. (2017)	Longitudinal case studies (V)	2	35.5 ± 7.8	2:0	Thoracic n = 2	Not reported	Not reported	ARKE 2.0	15 sessions of 30 min over a period of >4 weeks	Up time; walk time; walk distance; steps	None	Average across all sessions
Lester et al. (2018)	Pre-post case study (V)	1	21	1:0	Cervical	B, n = 1	‘since 2013’	Ekso GT	Three sessions over a 2 week period	Up time; walk time; stand-up time; steps; swing time	None	Each session
Okawara et al. (2020)	Single group pre-post intervention (IV)	20	43.3 ± 16.6	15:5	Cervical n = 10; Thoracic n = 9; Lumbar n = 1	A, n = 2 B, n = 4 C, n = 8 D, n = 6	80.4 ± 128.8	Hybrid Assistive Limb	20 sessions of 60 min 2–5 per week	Walk time; walk distance; walk speed; RPE	2MWT; 10MWT; TUG	First and last session for overall group; and average across all sessions overall and for each group
Piira et al. (2019)	Single blind pre-post RCT (I)	24	50.5 ± 6.4	9:10	Cervical n = 10; Thoracic n = 9	C, n = 6 D, n = 13	216 ± 50.9	Lokomat 4.0	59 ± 2 sessions of <90 min 3 per week for 6 months	Walk distance	6MWT; 10MWT	Average across all sessions
Rodriguez-Fernandez et al. (2025)	RCT crossover (II)	10	44.1 ± 5.93	9:1	Thoracic	A, n = 8 B, n = 2	126 ± 81	ABLE exo-skeleton	90 min per session 2 per week for 5 weeks	Up time; walk time; walk distance; steps; walk speed	6MWT; 10MWT; TUG	First and last sessions*
Stampacchia et al. (2020)	Comparative pre-post intervention (II)	52	49 ± 15.3	38:14	Cervical n = 11; Thoracic n = 23	A, n = 15 B, n = 9 C, n = 10 D, n = 18	Median 12 IQR 12–60	Lokomat or Ekso	20 sessions of FES and 20 sessions of RAGT Group A: 30 min FES; 3 per week for 14 weeks. 40–60 min RAGT Group B: 30 min FES. 50–68 min RAGT; 3 per week for 14 weeks	Walk time; walk speed; steps	6MWT; 10MWT; TUG	First and last session
Swank et al. (2020)	Retrospective comparative longitudinal study (III)	59	48.2 ± 16.4	37:22	Cervical n = 38; Thoracic n = 17; Lumbar n = 4	A, n = 2 B, n = 2 C, n = 21 D, n = 13 Non-traumatic SCI, n = 21	Not reported	Not reported	Not reported	Up time; walk time; steps; RAGT time per day	None	First, ninth and last session
Tsai et al. (2024)	RCT pilot study (I)	32	46.3 ± 18.3	19	9	AIS A, n = 7 AIS B, n = 6 AIS C, n = 15	≤3	Ekso GT	9 ± 4 sessions of 60 min 2–3 per week to discharge	Up time; walk time; steps; RPE	None	Average across all sessions
van Dijsseldonk et al. (2020)	Single group intervention (IV)	14	Median 29 IQR 24–49	7:7	Thoracic n = 14	A, n = 13 B, n = 1	Median 75 IQR 9–324	ReWalk Personal 6.0	9 ± 5 sessions over 2–3 weeks	Walk distance; steps; distance without rest; active time	None	Average across all sessions
Williams et al. (2021)	Parallel group RCT pilot study (II)	6	34.2 ± 10.8	5:0	Cervical n = 1; Thoracic n = 4	A, n = 3 B, n = 2	73.4 ± 120.5	Lokomat or Ekso	36 sessions of 45 min walking 3 per week for 12 weeks	Training duration; early/late speed; early/late steps; early/late distance; RPE	10WMT	Average across first five and last five sessions
Wirz et al. (2017)	RCT (II)	21	35.0 ± 0.9	16:2	C4-T12	B, n = 9 C, n = 9	1.3 ± 0.7	Lokomat	34 ± 1 sessions; 3–5 per week for 8 weeks Group A: ≥ 50 min walking per session Group B: ≤ 25 min walking per session	Walk speed; walk distance; session duration	None	Walk distance and duration average across all sessions Walk speed average during first and last week
Wright et al. (2023)	Quasi-experimental pre-post intervention (IV)	24	45 ± 12	17:7	Cervical n = 1; Thoracic n = 19; Lumbar n = 4	A, n = 9 B, n = 7 C, n = 1 D, n = 7	19.0 ± 45.7	ABLE exo-skeleton	10 ± 4 sessions of 60 min 3 per week for 4 weeks	Up time; walk time; steps; speed; distance; therapy time; RPE	6MWT; 10MWT; TUG	Second, sixth and 11th sessions
Wu et al. (2012)	RCT crossover (II)	10	47 ± 7	8:2	Cervical n = 8; Thoracic n = 2	D, n = 10	69.6 ± 45.6	Custom RAGT system	45 min sessions 3 per week for 8 weeks	Training time; distance; speed; BW support; stride and step length; cadence; single and double support; peak and rate of torque during flexion and extension of hip, knee and ankle	6MWT	First and last sessions

*=for primary outcomes only. 2MWT = 2-Minute Walk Test; 6MWT = 6-Minute Walk Test; 10MWT = 10-Metre Walk Test; ASIA: American Spinal Injury Association; BW: Bodyweight; FES: Functional Electrical Stimulation; IQR: Interquartile Range; METs: Metabolic Equivalents; MVPA: Moderate-Vigorous Physical Activity; RAGT: Robot-Assisted Gait Training; RCT: Randomised Controlled Trial; RoM: Range-of-Motio n; RPE: Rating of Perceived Exertion; TUG: Timed Up-and-Go.

Numerical data presented either as ‘n’ or mean ± SD unless otherwise stated. Sample size column reflects the total number of participants initially recruited to each study.

Thirteen of the included studies involved the use of an Ekso exoskeleton;^38,40–51^ five used a Lokomat device;^39,50–53^ four used a Hybrid Assistive Limb;^54–57^ three used the ReWalk;^45,58,59^ two used the ABLE exo-skeleton;^60,61^ and another two used the LEXO robotic gait trainer.^62,63^ One study did not report the name of the device,^64^ and the remaining three studies involved the use of a discrete exoskeleton.^65–67^

Information related to the training period indicated high variability in the protocol used: from a single training session^40,62^ to three sessions per week for six months.^52^ The most common training session duration was 60–90 min (n = 18; 60% studies), 3–5 times per week (n = 17; 57% studies), for 6–12 weeks (n = 10; 33% studies).

Table 2 summarises the risk of bias assessment conducted using the Physiotherapy Evidence Database scale for the randomised studies. Eligibility criteria were reported in all of the included randomised controlled trials and their overall methodological quality ranged from ‘fair’ (n = 5) to ‘good’ (n = 3), with the total scores ranging from 4/10^67^ to 8/10.^39^ Four studies received total scores of 5/10, and the remaining paper scored a total of 6/10.^53,61^

**Table 2.** Summary of risk of bias assessment for included randomised studies according to the 11 domains of the PEDro scale.

Study (date)	1. Eligibility criteria specified	2. Subjects were randomly allocated to groups (or to an order in which treatments were received in a crossover study)	3. Allocation was concealed	4. The groups were similar at baseline regarding the most important prognostic indicators	5. There was blinding of all subjects	6. There was blinding of all therapists who administered the therapy	7. There was blinding of all assessors who measured ≥1 key outcome	8. Measures of ≥1 key outcome were obtained from >85% of the subjects initially allocated to groups	9. All subjects for whom outcome measures were available received the treatment or control condition as allocated or, where this was not the case, data for ≥1 key outcome was analysed by ‘intention to treat’	10. The results of between-group statistical comparisons are reported for ≥1 key outcome	11. The study provides both point measures and measures of variability for ≥1 key outcome	Total score^†^ (/10)	Overall methodological quality*
Hong et al. (2020)	Y	Y	N	N	N	N	N	Y	Y	Y	Y	**5**	**Fair**
Lam et al. (2015)	Y	Y	N	Y	Y	N	Y	Y	Y	Y	Y	**8**	**Good**
Piira et al. (2019)	Y	Y	Y	N	N	N	Y	N	N	Y	Y	**5**	**Fair**
Rodriguez-Fernandez (2025)	Y	Y	N	Y	N	N	N	Y	Y	Y	Y	**6**	**Good**
Tsai et al. (2024)	Y	Y	Y	N	N	N	N	Y	N	Y	Y	**5**	**Fair**
Williams et al. (2021)	Y	Y	Y	Y	N	N	Y	N	Y	N	N	**5**	**Fair**
Wirz et al. (2017)	Y	Y	Y	Y	N	N	N	Y	N	Y	Y	**6**	**Good**
Wu et al. (2012)	Y	Y	N	N	N	N	N	N	Y	Y	Y	**4**	**Fair**

† Calculated by summing the points from items 2–11 (‘Y’ = 1 point, ‘N’ = 0 points); *total score <4 = ‘poor’, 4–5 = ‘fair’, 6–8 = ‘good’, 9–10 = ‘excellent’ (Cashin and McAuley, 2020).

**Figure 2.**
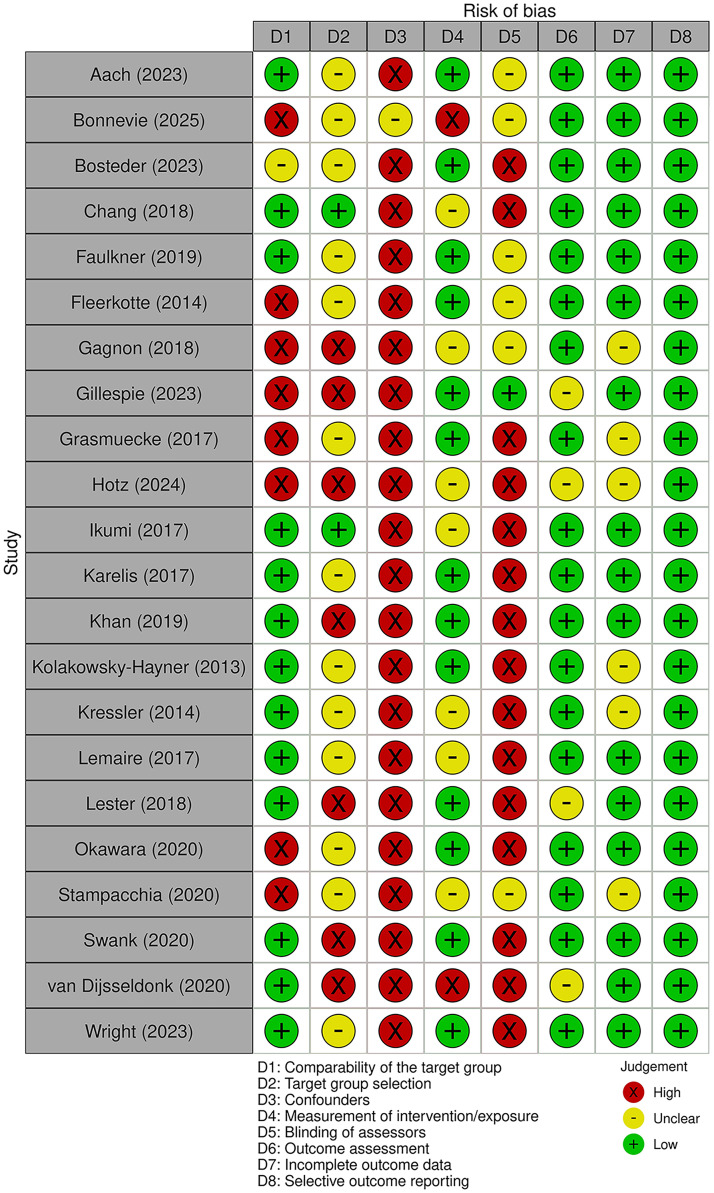
Risk of bias assessment results for non-randomised studies. Figure created using the Robvis tool.^36^

Figure 2 presents the results of the risk of bias assessment using the Revised Risk of Bias Assessment Tool for Nonrandomized Studies. 21/22 of the studies assessed with this tool were deemed to have a high risk of bias for the ‘Confounders’ domain, while all 22 had low risk of bias for the ‘Selective outcome reporting’ domain. There were mixed results for the remaining domains.

The data for physical activity outcomes reported by multiple studies at the beginning and end of the gait training can be found in Table 3. A list of other relevant physical activity outcomes that were recorded by only one study can be found within the supplementary materials (Supp 1). Relevant outcome data that were only reported as an overall average across the training period can also be found within the supplementary materials (Supp 2). Finally, the data for the secondary outcomes of interest (2-min walk test, 6-min walk test, 10-metre walk test, timed up-and-go test, and rating of perceived exertion) reported by multiple studies can be found in Supp 3.

**Table 3.** Mean physical activity outcome data from included studies presenting results from the start and end of robot-assisted gait training (RAGT).

		Up time (min)		Walk time (min)		Walk speed (m/s)		Walk distance (m)		Steps (*n*)	
Study (date)	Participants (*n*)	Start of RAGT	End of RAGT	Start of RAGT	End of RAGT	Start of RAGT	End of RAGT	Start of RAGT	End of RAGT	Start of RAGT	End of RAGT
Aach et al. (2023)	50			16.1 ± 5.7	28.9 ± 6.4 (*p* < 0.001)	0.25 ± 0.11	0.61 ± 0.19 (*p* < 0.001)	262 ± 181	997 ± 411 (*p* < 0.001)	30.0 ± 9.2	20.4 ± 5.0 (*p* < 0.05)
Chang et al. (2020)	1	29.8	39.7	2.6	23.7					111	946
Faulkner et al. (2021)	12	35 ± 14	48 ± 13.0 (*p* < 0.05; d = 0.95)	10.0 ± 3.0	24.0 ± 8.0 (*p* < 0.01; d = 2.47)					193 ± 47	523 ± 125 (*p* < 0.01; d = 3.51)
Fleerkotte et al. (2014)	10			14.5 ± 6.1	22.7 ± 18.2	0.49	0.56 (*p* = 0.015)				
Gagnon et al. (2018)	13	40.3 ± 0.6	53.4 ± 4.4	21.4 ± 0.8	40.3 ± 4.5					540 ± 35	1679 ± 168
Gillespie et al. (2023)	Baseline n = 99 Final n = 23	16.6 ± 6.2	22.9 ± 7.0	10.3 ± 5.4	19.6 ± 7.2					303 ± 176	664 ± 289
Grasmücke et al. (2017)	55			12.6 ± 4.7	29.6 ± 5.6 (*p* < 0.001)	0.24 ± 0.09	0.51 ± 0.18 (*p* < 0.001)		184.6 ± 134.7 (*p* < 0.001)		
Hong et al. (2020)	EKSO n = 22 ReWalk n = 28									EKSO: 547 ReWalk: 408	EKSO: 1601 ± 349 (*r*^2^ = 0.082, y = 16.62x + 1267.96) ReWalk: 1718 ± 731 (*r*^2^ = 0.0956, y = 27.90x + 931.24)
Hotz et al. (2024)	2	31.2 ± 12.4	41.5 ± 3.9			1.44 ± 1.19	2.47 ± 1.72	639.4 ± 681.5	1299.8 ± 989.6	1349.9 ± 1239.7	2510.5 ± 1270.3
Ikumi et al. (2017)	1			7.6	14.9			25.2	148.3		
Khan et al. (2019)	11					0.03	0.41	23.8	1102.9	68	2311
Kolakowsky-Hayner et al. (2013)	7	43.3 ± 8.3	57.3 ± 9.6	16.9 ± 3.9	34.2 ± 11.8			64.5 ± 32.8	237.7 ± 202.9		
Kressler et al. (2014)	3	55.4 ± 10.0	61.1 ± 2.4	41.1 ± 12.4	51.1 ± 8.1			171.2 ± 140.2	549.1 ± 394.0	549 ± 453	1582 ± 936
Lam et al. (2015)	13					Loko-R 0.33 ± 0.10 Control 0.44 ± 0.20	Loko-R 0.47 ± 0.06 Control 0.53 ± 0.08 Between groups *F*(1,11) = 23.1 (*p* = 0.001) Within groups *F*(1, 11) = 41.6 (*p* < 0.001)				
Lester et al. (2018)	1	19	31	7	17					83	589
Okawara et al. 2020)	20			30.0 ± 11.0	41.0 ± 2.0 (*p *< 0.01)	0.16 ± 0.06	0.19 ± 0.08 (*p* < 0.01)	300 ± 190	460 ± 210 (*p* < 0.01)		
Rodriguez-Fernandez et al. (2025)	10	28.3 ± 12.5	32.6 ± 12.1 (*p* < 0.001)	6.0 ± 6.9	15.8 ± 8.8 (*p* < 0.001)	0.09 ± 0.07	0.16 ± 0.08 (*p* < 0.001)	50.0 ± 107.7	188.3 ± 190.8 (*p* < 0.001)	170.4 ± 310.6	499.9 ± 429.3 (*p* < 0.001)
Stampacchia et al. (2020)	Group B n = 25					Group B 0.11 ± 0.04	Group B 0.15 ± 0.04 (*p* < 0.05)				
Swank et al. (2020)	59	16.7	31.3	8.5	28.3					8	29
Williams et al. (2021)	EKSO n = 3 Lokomat n = 2					Lokomat 0.55 ± 0.03	Lokomat 0.61 ± 0.09	Lokomat 1118.9 ± 200.4	Lokomat 1587.6 ± 384.38	EKSO 970 ± 264	EKSO 1736 ± 369
Wirz et al. (2017)	Long duration n = 9 Short duration n = 9					Long 0.51 ± 0.08 Short 0.51 ± 0.10 Between groups (*p* = 0.92)	Long 0.55 ± 0.07 Short 0.54 ± 0.12 Between groups (*p* = 0.866)				
Wright et al. (2023)	24	16.7	20.5	4.8 ± 6.5	12.4 ± 10.7 (*p* < 0.001)	0.10 ± 0.10	0.20 ± 0.10 (*p* < 0.001)	55.2 ± 111.9	157.8 ± 164.1 (*p* < 0.001)	139 ± 240	423 ± 416 (*p* < 0.001)
Wu et al. (2012)	8					0.67 ± 0.20	0.76 ± 0.23 (*p* = 0.03)	1680 ± 640	2270 ± 650		

Only outcomes that were assessed in more than one of the included studies have been presented.

Outcome data presented as mean ± SD where this information was provided in the source article. Statistical data presented where this information was provided in the source article.

All the studies that presented data for the primary outcomes of up time (n = 11), walk time (n = 15), walk speed (n = 13), walk distance (n = 12), and steps (n = 14) at the beginning and end of robot-assisted gait training found an overall increase across the period. Overall, the included studies provided very low certainty of evidence for each outcome due to serious concerns related to study limitations, indirectness, and imprecision (Table 4).

**Table 4.** Summary of certainty of evidence assessment for change in physical activity outcomes from beginning to end of RAGT.

Outcome	Effect over time	Number of participants (study design type)	Certainty of evidence^‡^ (GRADE assessment)
Up time (mins)	11/11 studies found an increase across the RAGT period. Of these, two indicated that this difference was significant (*p* < 0.05), while the others only provided descriptive statistics.	231 (1 randomised; 1 NRC*; 6 NRNC; 3 case reports/series)	Very low ⊕OOO (due to serious study limitations, indirectness, and imprecision)
Walk time (mins)	15/15 studies found an increase across the RAGT period. Of these, six indicated this difference was significant (*p* < 0.01), while the others only provided descriptive statistics.	365 (1 randomised; 1 NRC*; 9 NRNC*; 4 case reports/series)	Very low ⊕OOO (due to serious study limitations, indirectness, and imprecision)
Walk speed (m/s)	13/13 studies found an increase across the RAGT period. Of these, nine indicated that this difference was significant (*p* < 0.05), while the others only provided descriptive statistics.	248 (5 randomised^†^; 8 NRNC*)	Very low ⊕OOO (due to serious study limitations, indirectness, and imprecision)
Walk distance (m)	12/12 studies found an increase across the RAGT period. Of these, five indicated that this difference was significant (*p* < 0.01), while the others only provided descriptive statistics.	193 (3 randomised*; 7 NRNC; 2 case reports/series)	Very low ⊕OOO (due to serious study limitations, indirectness, and imprecision)
Steps (n)	13/14 studies found an increase across the RAGT period. Of these, three indicated that this difference was significant (*p* < 0.01), while the others only provided descriptive statistics for the effect over time. The remaining study found a significant decrease in steps across the RAGT period (*p* < 0.05).	338 (3 randomised*; 2 NRC*; 6 NRNC; 3 case reports/series)	Very low ⊕OOO (due to serious study limitations, indirectness, and imprecision)

GRADE: Grading of Recommendation, Assessment, Development and Evaluation; NRC: Non-randomised comparative; NRNC: Non-randomised non-comparative; RAGT: Robot-Assisted Gait Training. All GRADE assessments were conducted starting from a ‘low’ certainty of evidence due to the greater proportion of non-randomised trials and/or non-randomised participants involved for each outcome (Shao et al., 2023).

*Including one pilot study; ^†^including two pilot studies; ^‡^see Supp 5 for details.

Several studies also reported data collected during the following functional walk tests: 2-min walk test (n = 2), 6-min walk test (n = 9), 10-metre walk test (n = 13), and the timed up-and-go (n = 7) test. All studies observed improvements in each of the tests they measured, with the exception of the 10-metre walk test in the study by Piira et al.,^52^ who observed no pre- to post-training change. Overall, the included studies provided very low certainty of evidence for all functional walk tests due to serious concerns related to study limitations, indirectness, and imprecision (Supp 4). The underlying justifications for all ratings according to the Grading of Recommendations, Assessment, Development and Evaluation framework can be found in the supplementary materials (Supp 5 and Supp 6).

## Discussion

The purpose of this systematic review was to investigate the effect of robot-assisted gait training on physical activity outcomes for people with spinal cord injury. The main findings, based on 30 studies, showed it significantly improved physical activity outcomes over time. However, these findings were constrained by very low certainty evidence for the observed effect on up time, walk time, walk speed, walk distance, and steps. While the trends of the synthesised data were encouraging, research with more robust methodologies is urgently needed to improve confidence in the effectiveness of robot-assisted gait training on physical activity outcomes following spinal cord injury.

This was the first systematic review to focus primarily on physical activity outcomes during robot-assisted gait training sessions. All the included studies observed an improvement over time for all the synthesised physical activity outcomes, which is in line with the secondary findings of another systematic review that noted a trend of improvement over time in walk time, walk distance, and steps.^17^ This general increase in physical activity parameters is likely to have implications beyond the primary purpose of improving gait: physical, mental, and social wellbeing have all been demonstrated to be positively affected by increasing physical activity for patients following spinal cord injury.^68–72^ This is particularly important when considering that function, health, and relationships have previously been identified as key priorities for individuals following spinal cord injury,^73^ combined with the fact that a large proportion of people with spinal cord injury fail to meet established physical activity guidelines.^74,75^

Previous research has suggested optimal robot-assisted gait training protocols should involve 24–36 sessions across an 8–12 week period (i.e., 3 per week), with each involving 60 min of moderate-to-vigorous exercise to improve aerobic and functional capacity.^74,76,77^ The training protocols employed in the included studies of the present systematic review were largely in line with the aforementioned optimal session parameters; involving patients who underwent 60–90 min training sessions, 3–5 times per week, for a 6–12 week period. Such session parameters appear to be typical, since they were similar to with those reported in two other systematic reviews that investigated other aspects of robot-assisted gait training.^78,79^ This positive finding indicates that clinicians are largely aware of how best to prescribe training session frequencies within rehabilitation programs, though less is known about optimal intra-session variables such as device-related parameters.^77^

Although 11 of the included studies employed comparative designs, five compared different variables between groups of users each undergoing robot-assisted gait training: exoskeleton-applied resistance vs assistance;^39,67^ treadmill gait training vs overground gait training;^50^ Ekso device vs Lokomat device;^51^ and long training sessions vs short training sessions.^53^ The remaining six studies compared a group of patients using robot-assisted gait training against a ‘usual care’ group. However, none of them provided physical activity data for their respective control groups since differences in physical activity outcomes were not the primary comparison of interest.^42,45,52,64^ As a result, there is a clear lack of published research involving appropriate control groups to allow for a comprehensive investigation into the efficacy of robot-assisted gait training for improving physical activity outcomes following spinal cord injury. This, combined with the abovementioned issue of underpowered sample sizes, was a major factor in the eventual very low certainty of evidence ratings above.

Each outcome in the present review provided an insight into the effect of robot-assisted gait training on commonly measured spatiotemporal parameters of gait. However, several included studies also recorded additional kinetic and kinematic gait measures, finding improvements in some but not all of them (Supp 1). Since these additional parameters were each only measured in single studies, these data were not relevant for synthesis in the present review. However, they do suggest that the observed similarities in the direction of change for up time, walk time, walk distance, walk speed, and steps between studies does not imply a universal improvement in all aspects of gait over time. Therefore, while the results of this systematic review support the use of robot-assisted gait training as a means of improving physical activity outcomes, further research is required to fully understand its effect on gait as a whole and the implications this may have for people with spinal cord injury.

There are several limitations that should be considered when interpreting the findings of this systematic review. Firstly, meta-analysis was not possible due to study heterogeneity. Additionally, all eligible studies were included irrespective of demographic variables, clinical variables, robot-assisted gait training device, or the training protocol that was utilised. This introduced potential confounders that may have led to an over- or under-estimation of the effect for different outcomes.

All of the included studies had high risk of bias for at least one domain according to the relevant quality assessment score used. Several solely provided descriptive statistics while only two studies presented 95% confidence intervals for secondary outcomes, which was a major contributor to the overall very low certainty of evidence reported here.

19/30 reviewed studies were non-randomised and non-comparative while only eight studies involved a randomised design, which limits the ability for the true effect of robot-assisted gait training to be established relative to a comparable control. Furthermore, there were five pilot studies and six case reports while the sample sizes of the remaining studies were relatively small (range 5–99).

To conclude, robot-assisted gait training significantly improved physical activity measures, namely spatiotemporal gait parameters, over time during training for patients with spinal cord injury. The inclusion of robot-assisted gait training in rehabilitation for patients with spinal cord injury is therefore a useful adjunct that may have positive implications for the impact of greater independence and improved walking ability. However, there was very low certainty of evidence to support the synthesised outcomes. Future research is required to investigate the effects of potential confounders, and appropriately powered studies with high quality randomised designs would improve the overall certainty of evidence.

**Clinical messages**

- Robot-assisted gait training improves physical activity and functional outcomes after spinal cord injury.
- Such improvements have known positive physical, mental, and social effects for people with spinal cord injury.
- Robot-assisted gait training programmes should involve 3–5 sessions/week for 6–12 weeks, each comprising 60–90 min of moderate-to-vigorous exercise.

## Supplemental Material

sj-docx-1-cre-10.1177_02692155251411864 - Supplemental material for The effect of robot-assisted gait training on physical activity outcomes in people with spinal cord injury: A systematic review Supplemental material, sj-docx-1-cre-10.1177_02692155251411864 for The effect of robot-assisted gait training on physical activity outcomes in people with spinal cord injury: A systematic review by James Belsey, Andrew Reid, Scott Hannah, Louise Johnson and James Faulkner in Clinical Rehabilitation

sj-docx-2-cre-10.1177_02692155251411864 - Supplemental material for The effect of robot-assisted gait training on physical activity outcomes in people with spinal cord injury: A systematic review Supplemental material, sj-docx-2-cre-10.1177_02692155251411864 for The effect of robot-assisted gait training on physical activity outcomes in people with spinal cord injury: A systematic review by James Belsey, Andrew Reid, Scott Hannah, Louise Johnson and James Faulkner in Clinical Rehabilitation

sj-docx-3-cre-10.1177_02692155251411864 - Supplemental material for The effect of robot-assisted gait training on physical activity outcomes in people with spinal cord injury: A systematic review Supplemental material, sj-docx-3-cre-10.1177_02692155251411864 for The effect of robot-assisted gait training on physical activity outcomes in people with spinal cord injury: A systematic review by James Belsey, Andrew Reid, Scott Hannah, Louise Johnson and James Faulkner in Clinical Rehabilitation

sj-docx-4-cre-10.1177_02692155251411864 - Supplemental material for The effect of robot-assisted gait training on physical activity outcomes in people with spinal cord injury: A systematic review Supplemental material, sj-docx-4-cre-10.1177_02692155251411864 for The effect of robot-assisted gait training on physical activity outcomes in people with spinal cord injury: A systematic review by James Belsey, Andrew Reid, Scott Hannah, Louise Johnson and James Faulkner in Clinical Rehabilitation

sj-docx-5-cre-10.1177_02692155251411864 - Supplemental material for The effect of robot-assisted gait training on physical activity outcomes in people with spinal cord injury: A systematic review Supplemental material, sj-docx-5-cre-10.1177_02692155251411864 for The effect of robot-assisted gait training on physical activity outcomes in people with spinal cord injury: A systematic review by James Belsey, Andrew Reid, Scott Hannah, Louise Johnson and James Faulkner in Clinical Rehabilitation

sj-docx-6-cre-10.1177_02692155251411864 - Supplemental material for The effect of robot-assisted gait training on physical activity outcomes in people with spinal cord injury: A systematic review Supplemental material, sj-docx-6-cre-10.1177_02692155251411864 for The effect of robot-assisted gait training on physical activity outcomes in people with spinal cord injury: A systematic review by James Belsey, Andrew Reid, Scott Hannah, Louise Johnson and James Faulkner in Clinical Rehabilitation

## References

[1] Lu Y Shang Z Zhang W , et al. Global incidence and characteristics of spinal cord injury since 2000–2021: a systematic review and meta-analysis. BMC Med 2024; 22: 285. 38972971 10.1186/s12916-024-03514-9 PMC11229207

[2] Ho J-W Ko K-Y Law SW , et al. The effectiveness of robotic-assisted upper limb rehabilitation to improve upper limb function in patients with cervical spinal cord injuries: a systematic literature review. Front Neurol 2023; 14: 1126755. 37621855 10.3389/fneur.2023.1126755 PMC10445651

[3] Morone G Pirrera A Iannone A , et al. Development and use of assistive technologies in spinal cord injury: a narrative review of reviews on the evolution, opportunities, and bottlenecks of their integration in the health domain. Healthcare (Switzerland) 2023; 11: 1646. 10.3390/healthcare11111646 PMC10252185 37297786

[4] Naka T Hayashi T Sugyo A , et al. Effect of age at injury on walking ability following incomplete cervical spinal cord injury: a retrospective cohort study. Spine Surg Relat Res 2022; 6: 604–609. 36561160 10.22603/ssrr.2021-0240 PMC9747207

[5] Sinovas-Alonso I Gil-Agudo Á Cano-De-la-cuerda R , et al. Walking ability outcome measures in individuals with spinal cord injury: a systematic review. Int J Environ Res Public Health 2021; 18: 9517. 34574443 10.3390/ijerph18189517 PMC8472084

[6] Maggioni S Lünenburger L Riener R , et al. Assessing walking ability using a robotic gait trainer: opportunities and limitations of assist-as-needed control in spinal cord injury. J Neuroeng Rehabil 2023; 20: 21. 37735690 10.1186/s12984-023-01226-4 PMC10515081

[7] Diop M Epstein D . A systematic review of the impact of spinal cord injury on costs and health-related quality of life. Pharmacoecon Open 2024; 8: 793–808. 39150624 10.1007/s41669-024-00517-3 PMC11499558

[8] Zhang L Wang Y Ye T , et al. Quality of clinical practice guidelines relevant to rehabilitation of knee osteoarthritis: a systematic review. Clin Rehabil 2023; 37: 986–1008. 36540949 10.1177/02692155221144892

[9] Arroyo-Fernández R Menchero-Sánchez R Pozuelo-Carrascosa DP , et al. Effectiveness of body weight-supported gait training on gait and balance for motor-incomplete spinal cord injuries: a systematic review with meta-analysis. J Clin Med 2024; 13: 1105. 38398415 10.3390/jcm13041105 PMC10888564

[10] La Rosa G Avola M Di Gregorio T , et al. Gait recovery in spinal cord injury: a systematic review with metanalysis involving new rehabilitative technologies. Brain Sci 2023; 13: 03. 10.3390/brainsci13050703 PMC10216369 37239175

[11] Fabbri I Betti F Tedeschi R . Gait quality after robot therapy compared with physiotherapy in the patient with incomplete spinal cord injured: a systematic review. eNeurologicalSci 2023; 31: 100467. 37304729 10.1016/j.ensci.2023.100467 PMC10248036

[12] Patathong T Klaewkasikum K Woratanarat P , et al. The efficacy of gait rehabilitations for the treatment of incomplete spinal cord injury: a systematic review and network meta-analysis. J Orthop Surg Res 2023; 18: 60. 36683024 10.1186/s13018-022-03459-w PMC9869518

[13] Stampacchia G Gazzotti V Olivieri M , et al. Gait robot-assisted rehabilitation in persons with spinal cord injury: a scoping review. NeuroRehabilitation 2022; 51: 609–647. 36502343 10.3233/NRE-220061

[14] Aguirre-Güemez AV Pérez-Sanpablo AI Quinzaños-Fresnedo J , et al. Walking speed is not the best outcome to evaluate the effect of robotic assisted gait training in people with motor incomplete spinal cord injury: a systematic review with meta-analysis. J Spinal Cord Med 2019; 42: 142–154. 29065788 10.1080/10790268.2017.1390644 PMC6419626

[15] Nam KY Kim HJ Kwon BS , et al. Robot-assisted gait training (Lokomat) improves walking function and activity in people with spinal cord injury: a systematic review. J Neuroeng Rehabil 2017; 14: 24. 28330471 10.1186/s12984-017-0232-3 PMC5363005

[16] Lefeber N Swinnen E Kerckhofs E . The immediate effects of robot-assistance on energy consumption and cardiorespiratory load during walking compared to walking without robot-assistance: a systematic review. Disabil Rehabil Assist Technol 2017; 12: 657–671. 27762641 10.1080/17483107.2016.1235620

[17] Duddy D Doherty R Connolly J , et al. The effects of powered exoskeleton gait training on cardiovascular function and gait performance: a systematic review. Sensors 2021; 21: 3207. 34063123 10.3390/s21093207 PMC8124924

[18] Hayes SC James WCR Forbes White HS , et al. The effects of robot assisted gait training on temporal-spatial characteristics of people with spinal cord injuries: a systematic review. Journal of Spinal Cord Medicine 2018; 41: 529–543. 29400988 10.1080/10790268.2018.1426236 PMC6117598

[19] Bin L Wang X Jiatong H , et al. The effect of robot-assisted gait training for patients with spinal cord injury: a systematic review and meta-analysis. Front Neurosci 2023; 17: 1252651. 37680972 10.3389/fnins.2023.1252651 PMC10482434

[20] Fang CY Tsai JL Li GS , et al. Effects of robot-assisted gait training in individuals with spinal cord injury: a meta-analysis. Biomed Res Int 2020; 2020: 2102785. 32280681 10.1155/2020/2102785 PMC7115057

[21] Alashram AR Annino G Padua E . Robot-assisted gait training in individuals with spinal cord injury: a systematic review for the clinical effectiveness of Lokomat. J Clin Neurosci 2021; 91: 260–269. 34373038 10.1016/j.jocn.2021.07.019

[22] Miller SA Forrest JL . Enhancing your practice through evidence-based decision making: PICO, learning how to ask good questions. J Evid-Based Dental Pract 2001; 1: 136–141.

[23] Page MJ McKenzie JE Bossuyt PM , et al. The PRISMA 2020 statement: an updated guideline for reporting systematic reviews. The BMJ 2021; 372: 71. 10.1136/bmj.n71 PMC8005924 33782057

[24] Page MJ Moher D Bossuyt PM , et al. PRISMA 2020 Explanation and elaboration: updated guidance and exemplars for reporting systematic reviews. The BMJ 2021; 372: n160. 10.1136/bmj.n160 PMC8005925 33781993

[25] Campbell M McKenzie JE Sowden A , et al. Synthesis without meta-analysis (SWiM) in systematic reviews: reporting guideline. The BMJ 2020; 368: 16890. 10.1136/bmj.l6890 PMC7190266 31948937

[26] Murad MH Mustafa RA Schünemann HJ , et al. Rating the certainty in evidence in the absence of a single estimate of effect. Evid Based Med 2017; 22: 85–87. 28320705 10.1136/ebmed-2017-110668 PMC5502230

[27] Shao SC Kuo LT Huang YT , et al. Using grading of recommendations assessment, development, and Evaluation (GRADE) to rate the certainty of evidence of study outcomes from systematic reviews: A quick tutorial. Dermatologica Sinica 2023; 41: 3–7.

[28] Guyatt GH Oxman AD Schünemann HJ , et al. GRADE Guidelines: a new series of articles in the Journal of Clinical Epidemiology. J Clin Epidemiol 2011; 64: 380–382. 21185693 10.1016/j.jclinepi.2010.09.011

[29] Jetté M Sidney K Blümchen G . Metabolic equivalents (METS) in exercise testing, exercise prescription, and evaluation of functional capacity. Clin Cardiol 1990; 13: 555–565. 2204507 10.1002/clc.4960130809

[30] Franklin BA Brinks J Berra K , et al. Using metabolic equivalents in clinical practice. Am J Cardiol 2018; 121: 382–387. 29229271 10.1016/j.amjcard.2017.10.033

[31] Moeyaert M Maggin D Verkuilen J . Reliability, validity, and usability of data extraction programs for single-case research designs. Behav Modif 2016; 40: 874–900. 27126988 10.1177/0145445516645763

[32] Drevon D Fursa SR Malcolm AL . Intercoder reliability and validity of WebPlotDigitizer in extracting graphed data. Behav Modif 2017; 41: 323–339. 27760807 10.1177/0145445516673998

[33] de Morton N . The PEDro scale is a valid measure of the methodological quality of clinical trials: a demographic study. Australian Journal of Physiotherapy 2009; 55: 129–133. 19463084 10.1016/s0004-9514(09)70043-1

[34] Moseley AM Rahman P Wells GA , et al. Agreement between the Cochrane risk of bias tool and physiotherapy evidence database (PEDro) scale: a meta-epidemiological study of randomized controlled trials of physical therapy interventions. PLoS One 2019; 14: e0222770. 10.1371/journal.pone.0222770 PMC6752782 31536575

[35] Seo HJ Kim SY Lee YJ , et al. RoBANS 2: a revised risk of bias assessment tool for nonrandomized studies of interventions. Korean J Fam Med 2023; 44: 249–260. 37423253 10.4082/kjfm.23.0034 PMC10522469

[36] McGuinness LA Higgins JPT . Risk-of-bias VISualization (robvis): an R package and Shiny web app for visualizing risk-of-bias assessments. Res Synth Methods [Internet] 2020; 12: 55–61. 32336025 10.1002/jrsm.1411

[37] Hohmann E Feldman M Hunt TJ , et al. Research pearls: how do we establish the level of evidence? Arthroscopy - J Arthroscopic Related Surg 2018; 34: 3271–3277. 10.1016/j.arthro.2018.10.002 30509436

[38] Tsai CY Weinrauch WJ Manente N , et al. Exoskeletal-Assisted walking during acute inpatient rehabilitation enhances recovery for persons with spinal cord injury—A pilot randomized controlled trial. J Neurotrauma 2024; 41: 2089–2100. 38661533 10.1089/neu.2023.0667 PMC11971545

[39] Lam T Pauhl K Ferguson A , et al. Training with robot-applied resistance in people with motor-incomplete spinal cord injury: pilot study. J Rehabil Res Dev 2015; 52: 113–130. 26230667 10.1682/JRRD.2014.03.0090

[40] Bosteder KD Moore A Weeks A , et al. Intensity of overground robotic exoskeleton training in two persons with motor-complete tetraplegia: a case series. Spinal Cord Ser Cases 2023; 9: 24. 10.1038/s41394-023-00584-4 PMC10313748 37391401

[41] Chang SH Zhu F Patel N , et al. Combining robotic exoskeleton and body weight unweighing technology to promote walking activity in tetraplegia following SCI: a case study. J Spinal Cord Med 2020; 43: 126–129. 30335593 10.1080/10790268.2018.1527078 PMC7006789

[42] Faulkner J Martinelli L Cook K , et al. Effects of robotic-assisted gait training on the central vascular health of individuals with spinal cord injury: a pilot study. J Spinal Cord Med 2021; 44: 299–305. 31525137 10.1080/10790268.2019.1656849 PMC7952073

[43] Gagnon DH Escalona MJ Vermette M , et al. Locomotor training using an overground robotic exoskeleton in long-term manual wheelchair users with a chronic spinal cord injury living in the community: lessons learned from a feasibility study in terms of recruitment, attendance, learnability, performance and safety. J Neuroeng Rehabil 2018; 15: 12. 29490678 10.1186/s12984-018-0354-2 PMC5831695

[44] Gillespie J Arnold D Trammell M , et al. Utilization of overground exoskeleton gait training during inpatient rehabilitation: a descriptive analysis. J Neuroeng Rehabil 2023; 20: 102. 37542322 10.1186/s12984-023-01220-w PMC10401799

[45] Hong EK Gorman PH Forrest GF , et al. Mobility skills with exoskeletal-assisted walking in persons with SCI: results from a three center randomized clinical trial. Front Robot AI 2020; 7: 93. 33501260 10.3389/frobt.2020.00093 PMC7805715

[46] Karelis AD Carvalho LP Castillo MJE , et al. Effect on body composition and bone mineral density of walking with a robotic exoskeleton in adults with chronic spinal cord injury. J Rehabil Med 2017; 49: 84–87. 27973679 10.2340/16501977-2173

[47] Kolakowsky-Hayner SA . Safety and feasibility of using the EksoTM bionic exoskeleton to aid ambulation after Spinal cord injury. J Spine 2013; S4: 8.

[48] Kressler J Thomas CK Field-Fote EC , et al. Understanding therapeutic benefits of overground bionic ambulation: exploratory case series in persons with chronic, complete spinal cord injury. Arch Phys Med Rehabil 2014; 95: 1878–1887.e4. 24845221 10.1016/j.apmr.2014.04.026

[49] Lester RM Gorgey AS . Feasibility of robotic exoskeleton ambulation in a C4 person with incomplete spinal cord injury: a case report. Spinal Cord Ser Cases 2018; 4: 36. 10.1038/s41394-018-0053-z PMC5947854 29736262

[50] Stampacchia G Olivieri M Rustici A , et al. Gait rehabilitation in persons with spinal cord injury using innovative technologies: an observational study. Spinal Cord 2020; 58: 988–997. 32251368 10.1038/s41393-020-0454-2

[51] Williams AMM Deegan E Walter M , et al. Exoskeleton gait training to improve lower urinary tract function in people with motor-complete spinal cord injury: a randomized pilot trial. J Rehabil Med 2021; 53: jrm00222. 10.2340/16501977-2864 PMC8638733 34383958

[52] Piira A Lannem AM Sørensen M , et al. Robot-assisted locomotor training did not improve walking function in patients with chronic incomplete spinal cord injury: a randomized clinical trial. J Rehabil Med 2019; 51: 385–389. 30895326 10.2340/16501977-2547

[53] Wirz M MacH O Maier D , et al. Effectiveness of automated locomotor training in patients with acute incomplete spinal cord injury: a randomized, controlled, multicenter trial. J Neurotrauma 2017; 34: 1891–1896. 27750478 10.1089/neu.2016.4643

[54] Grasmücke D Zieriacks A Jansen O , et al. Against the odds: what to expect in rehabilitation of chronic spinal cord injury with a neurologically controlled Hybrid Assistive Limb exoskeleton. A subgroup analysis of 55 patients according to age and lesion level. Neurosurg Focus 2017; 42: E15. 10.3171/2017.2.FOCUS171 28463613

[55] Ikumi A Kubota S Shimizu Y , et al. Decrease of spasticity after hybrid assistive limb® training for a patient with C4 quadriplegia due to chronic SCI. J Spinal Cord Med 2017; 40: 573–578. 27762171 10.1080/10790268.2016.1225913 PMC5815155

[56] Okawara H Sawada T Matsubayashi K , et al. Gait ability required to achieve therapeutic effect in gait and balance function with the voluntary driven exoskeleton in patients with chronic spinal cord injury: a clinical study. Spinal Cord 2020; 58: 520–527. 31831847 10.1038/s41393-019-0403-0

[57] Aach M Schildhauer TA Zieriacks A , et al. Feasibility, safety, and functional outcomes using the neurological controlled Hybrid Assistive Limb exoskeleton (HAL®) following acute incomplete and complete spinal cord injury – results of 50 patients. J Spinal Cord Med 2023; 46: 574–581. 37083596 10.1080/10790268.2023.2200362 PMC10274525

[58] Khan AS Livingstone DC Hurd CL , et al. Retraining walking over ground in a powered exoskeleton after spinal cord injury: a prospective cohort study to examine functional gains and neuroplasticity. J Neuroeng Rehabil 2019; 16: 145. 31752911 10.1186/s12984-019-0585-x PMC6868817

[59] van Dijsseldonk RB van Nes IJW Geurts ACH , et al. Exoskeleton home and community use in people with complete spinal cord injury. Sci Rep 2020; 10: 15600. 32973244 10.1038/s41598-020-72397-6 PMC7515902

[60] Wright MA Herzog F Mas-Vinyals A , et al. Multicentric investigation on the safety, feasibility and usability of the ABLxfE lower-limb robotic exoskeleton for individuals with spinal cord injury: a framework towards the standardisation of clinical evaluations. J Neuroeng Rehabil 2023; 20: 45. 37046307 10.1186/s12984-023-01165-0 PMC10091314

[61] Rodríguez-Fernández A Lobo-Prat J Toira-Campanya M , et al. Randomized, crossover clinical trial on the safety, feasibility, and usability of the ABLE exoskeleton: a comparative study with knee-ankle-foot orthoses. PLoS One 2025; 20: e0318039. 10.1371/journal.pone.0318039 PMC12112281 40424335

[62] Bonnevie T Moily K Barnes S , et al. People with spinal cord injury or stroke are able to reach moderate-to-vigorous intensity while exercising on an end-effector robot assisted gait trainer: a pilot study. J Rehabil Assist Technol Eng 2025; 12: 20556683241310865. 39790638 10.1177/20556683241310865 PMC11707787

[63] Hotz I Mildner S Stampfer-Kountchev M , et al. Robot-assisted gait training in patients with various neurological diseases: a mixed methods feasibility study. PLoS One 2024; 19: e0307434. 10.1371/journal.pone.0307434 PMC11349200 39190743

[64] Swank C Trammell M Bennett M , et al. The utilization of an overground robotic exoskeleton for gait training during inpatient rehabilitation - single-center retrospective findings. Int J Rehabil Res 2020; 43: 206–213. 32282573 10.1097/MRR.0000000000000409

[65] Fleerkotte BM Koopman B Buurke JH , et al. The effect of impedance-controlled robotic gait training on walking ability and quality in individuals with chronic incomplete spinal cord injury: an explorative study. J Neuroeng Rehabil 2014; 11: 26. 24594284 10.1186/1743-0003-11-26 PMC3975927

[66] Lemaire ED Smith AJ Herbert-Copley A , et al. Lower extremity robotic exoskeleton training: case studies for complete spinal cord injury walking. NeuroRehabilitation 2017; 41: 97–103. 28505991 10.3233/NRE-171461

[67] Wu M Landry JM Schmit B , et al. Robotic resistance treadmill training improves locomotor robotic resistance treadmill training improves locomotor function in human spinal cord injury: a pilot study function in human spinal cord injury: a pilot study recommended citation recommended citation. Arch Phys Med Rehabil [Internet] 2012; 93: 782–789. 22459697 10.1016/j.apmr.2011.12.018

[68] Itodo OA Flueck JL Raguindin PF , et al. Physical activity and cardiometabolic risk factors in individuals with spinal cord injury: a systematic review and meta-analysis. Eur J Epidemiol 2022; 37: 335–365. 35391647 10.1007/s10654-022-00859-4 PMC9187578

[69] Kawanishi CY Greguol M . Physical activity, quality of life, and functional autonomy of adults with spinal cord injuries. Adapted Phy Activity Quart [Internet] 2013; 30: 317–337. 10.1123/apaq.30.4.317 24197622

[70] Ginis KAM Jetha A MacK DE , et al. Physical activity and subjective well-being among people with spinal cord injury: a meta-analysis. Spinal Cord 2010; 48: 65–72. 19581918 10.1038/sc.2009.87

[71] Ponzano M Buren R Adams NT , et al. Effect of exercise on mental health and health-related quality of life in adults with spinal cord injury: a systematic review and meta-analysis. Arch Phys Med Rehabil 2024; 105: 2350–2361. 38556188 10.1016/j.apmr.2024.02.737

[72] Todd KR Lawrason SVC Shaw RB , et al. Physical activity interventions, chronic pain, and subjective well-being among persons with spinal cord injury: a systematic scoping review. Spinal Cord 2021; 59: 93–104. 32948846 10.1038/s41393-020-00550-z

[73] Simpson LA Eng JJ Hsieh JTC , et al. The health and life priorities of individuals with spinal cord injury: a systematic review. J Neurotrauma 2012; 29: 1548–1555. 22320160 10.1089/neu.2011.2226 PMC3501530

[74] Lawrason SVC Todd KR Shaw RB , et al. Physical activity among individuals with spinal cord injury who ambulate: a systematic scoping review. Spinal Cord 2020; 58: 735–745. 32322042 10.1038/s41393-020-0460-4

[75] Stendell L Stubbs PW Rivera E , et al. Are middle- or older-aged adults with a spinal cord injury engaging in leisure-time physical activity? A systematic review and meta-analysis. Arch Rehabil Res Clin Transl. 2024; 6: 100335. 39006108 10.1016/j.arrct.2024.100335 PMC11240020

[76] Hicks AL Martin Ginis KA Pelletier CA , et al. The effects of exercise training on physical capacity, strength, body composition and functional performance among adults with spinal cord injury: a systematic review. Spinal Cord 2011; 49: 1103–1127. 21647163 10.1038/sc.2011.62

[77] Nepomuceno P Souza WH Pakosh M , et al. Exoskeleton-based exercises for overground gait and balance rehabilitation in spinal cord injury: a systematic review of dose and dosage parameters. J Neuroeng Rehabil 2024; 21: 73. 38705999 10.1186/s12984-024-01365-2 PMC11070073

[78] Miller LE Zimmermann AK Herbert WG . Clinical effectiveness and safety of powered exoskeleton-assisted walking in patients with spinal cord injury: systematic review with meta-analysis. Medical Devices: Evidence and Research 2016; 9: 455–466. 27042146 10.2147/MDER.S103102 PMC4809334

[79] Shackleton C Evans R Shamley D , et al. Effectiveness of over-ground robotic locomotor training in improving walking performance, cardiovascular demands, secondary complications and user-satisfaction in individuals with spinal cord injuries: a systematic review. J Rehabil Med 2019; 51: 723–733. 31511902 10.2340/16501977-2601

