# Supplementary material for: The effect of robot-assisted gait training on physical activity outcomes in people with spinal cord injury: A systematic review
Source: Clin Rehabil. 2026 Feb 18;40(6):734–56. doi: 10.1177/02692155251411864 (PMC13191083; doi:10.1177/02692155251411864)
Supplement: sj-docx-1-cre-10.1177_02692155251411864 - Supplemental material for The effect of robot-assisted gait training on physical activity outcomes in people with spinal cord injury: A systematic review [file sj-docx-1-cre-10.1177_02692155251411864.docx]

Supp 1: Relevant physical activity outcomes that were recorded by only one of the included studies

| **Study**  **(date)** | **Outcomes** |
| --- | --- |
| Bonnevie  (2025) | Cumulative step count |
| Bosteder et al.  (2023) ^†^ | MET’s; MET’s during MVPA; duration of MVPA; % of session in MVPA |
| Chang et al.  (2020) | Steps/second* |
| Fleerkotte et al.  (2014) | **Cycle time**; **step symmetry**; **step length**; step width^‡^; **relative stance phase duration**; maximum knee flexion during stance phase^‡^; **RoM of** **hip** and knee^‡^ during stance phase |
| Khan et al.  (2019) | Steps per bout of walking without stopping* |
| Lester et al.  (2018) ^†^ | Swing time |
| van Dijsseldonk et al.  (2020) ^†^ | Distance covered without rest |
| Wu et al.  (2012) | Fast gait speed^‡^; **cadence**; **single support**; double support^‡^; **stride length**;  peak torque of hip^‡^, knee^‡^, and ankle^‡^ during flexion and extension;  rate of torque during flexion and extension of knee^‡^ and ankle^‡^;  rate of torque during hip flexion^‡^ and **hip** **extension** |
| ^†^Not enough data recorded or reported to allow for analysis of change in outcomes over time.  *Observed increase in outcome from start to end of RAGT period but no statistical information reported.  ^‡^Difference observed from start to end of RAGT period but not statistically significant (p>0.05).  **Bold text** = Statistically significant difference reported from start to end of RAGT period (p<0.05).  10MWT = 10-Metre Walk Test; MET = Metabolic Equivalent; MVPA = Moderate-Vigorous Physical Activity;  RAGT = Robot-Assisted Gait Training; RoM = Range-of-Motion. | |
