# Supplementary material for: The effect of robot-assisted gait training on physical activity outcomes in people with spinal cord injury: A systematic review
Source: Clin Rehabil. 2026 Feb 18;40(6):734–56. doi: 10.1177/02692155251411864 (PMC13191083; doi:10.1177/02692155251411864)
Supplement: sj-docx-2-cre-10.1177_02692155251411864 - Supplemental material for The effect of robot-assisted gait training on physical activity outcomes in people with spinal cord injury: A systematic review [file sj-docx-2-cre-10.1177_02692155251411864.docx]

Supp 2: Mean physical activity outcome data per RAGT session from included studies

| **Study**  **(date)** | **Participants (*n*)** | **Up time (min)** | **Walk time (min)** | **Walk speed (m/s)** | **Walk distance (m)** | **Steps (*n*)** |
| --- | --- | --- | --- | --- | --- | --- |
| Bosteder et al.  (2023) | 2 | 40 ± 3.3 | 34.7 ± 8.2 |  |  | 1035 ± 17 |
| Karelis et al.  (2017) | 5 | 48.4 ± 7.4 | 27 ± 5.4 |  |  | 904 ± 260 |
| Lemaire et al.  (2017) | 2 | 20.8 ± 1.1 | 9.2 ± 2.1 |  | 52.2 ± 24.1 | 222 ± 91.5 |
| Okawara et al.  (2020) | Overall group  n=20  High ability group  n=12  Low ability group  n=8 |  | Overall group  39.4 ± 2.2  High ability  40.8 ± 1.6  Low ability  37.2 ± 1.1  (p<0.01 between High & Low ability) | Overall group  0.18 ± 0.07  High ability  0.2 ± 0.08  Low ability  0.14 ± 0.00  (p=0.03 between High & Low ability) | Overall group  430 ± 190  High ability  500 ± 220  Low ability  310 ± 10  (p<0.01 between High & Low ability) |  |
| Piira et al.  (2019) | 19 |  |  |  | 2271 ± 465 |  |
| Tsai et al.  (2024) | 32 | 34.3 ± 9.4 | 25.4 ± 7.7 |  |  | 536 ± 157 |
| van Dijsseldonk et al. (2020)* | 14 |  | 50.4 ± 21.2 |  | 341.1 ± 333.6 | 5678 ± 7594 |
| Wirz et al.  (2017) | Long duration  n=9  Short duration  n=9 |  |  |  | Long duration  1647 ± 296.8  Short duration  803.8 ± 167.7  (p<0.01) |  |
| RAGT = Robot-Assisted Gait Training  *Data presented represent the average of each outcome aggregated across the whole RAGT period rather than for each session | | | | | | |
