# Supplementary material for: The effect of robot-assisted gait training on physical activity outcomes in people with spinal cord injury: A systematic review
Source: Clin Rehabil. 2026 Feb 18;40(6):734–56. doi: 10.1177/02692155251411864 (PMC13191083; doi:10.1177/02692155251411864)
Supplement: sj-docx-3-cre-10.1177_02692155251411864 - Supplemental material for The effect of robot-assisted gait training on physical activity outcomes in people with spinal cord injury: A systematic review [file sj-docx-3-cre-10.1177_02692155251411864.docx]

Supp 3: Secondary outcomes from included studies before and after Robot-Assisted Gait Training (RAGT)

|  | **2-minute walk test**  **(m)** | | **6-minute walk test**  **(m)** | | **10-metre walk test**  **(m/s)** | | **Timed up-and-go test**  **(s)** | | **Rating of perceived exertion**  **(0-10)** | |
| --- | --- | --- | --- | --- | --- | --- | --- | --- | --- | --- |
| **Study (date)** | Pre-RAGT | Post-RAGT | Pre-RAGT | Post-RAGT | Pre-RAGT | Post-RAGT | Pre-RAGT | Post-RAGT | Pre-RAGT | Post-RAGT |
| Aach  et al. (2023) |  |  | 135.6 ± 93.8 | 233.3 ± 131.0  (p<0.05) | 0.16 ± 0.21 | 0.37 ± 0.28  (p<0.001) | 56.3 ± 25.8 | 28.8 ± 20.5  (p<0.05) |  |  |
| Fleerkotte  et al. (2014) |  |  | 184.4 | 212.9  (p=0.005) | 0.61 | 0.67  (p=0.08) | 24.7 | 20.5  (p=0.012) |  |  |
| Gagnon  et al. (2018) |  |  |  |  | 0.15 ± 0.02 | 0.25 ± 0.05  (p<0.001) |  |  |  |  |
| Grasmücke  et al. (2017) |  |  | 97.8 ± 95.8 | 146.3 ± 118.1  (p<0.001) | 0.14 ± 0.16 | 0.28 ± 0.32  (p<0.001) |  |  |  |  |
| Hong  et al. (2020) |  |  | Overall group  99.8 ± 35.1  EKSO  74.8 ± 18.0  ReWalk 119.5 ± 32.7 | Overall group  125.3 ± 40.4  EKSO  96.8 ± 28.9  ReWalk  148.4 ± 33.0  Time effect  F(1.849, 88.734) =34.830 (p<0.0005) | Overall group  0.26 ± 0.07  EKSO  0.21 ± 0.06  ReWalk  0.36 ± 0.09 | Overall group  0.31 ± 0.09  EKSO  0.27 ± 0.08  ReWalk  0.42 ± 0.1  Time effect  F(1.841, 88.372) =13.921  (p<0.0005) | EKSO  90.3 ± 18.3  ReWalk  67.2 ± 15.3 | EKSO  72.2 ± 20.5  ReWalk  53.4 ± 11.2  Time effect  F(1.597, 68.665) =13.749  (p < 0.0005) |  |  |
| Hotz  et al. (2024) |  |  | 234.0 ± 274.3 | 269.0 ± 250.3 | 0.80 ± 0.85 | 1.05 ± 0.97 | 25.4 ± 22.3 | 14.5 ± 9.3 |  |  |
| Kressler  et al. (2014) | 8.8 ± 6.4 | 19.4 ± 12.3 |  |  | 0.07 ± 0.06 | 0.19 ± 0.13 |  |  |  |  |
| Only outcomes that were assessed in more than one of the included studies have been presented.  Outcome data presented as mean ± SD where this information was provided in the source article. Statistical data presented where this information was provided in the source article. | | | | | | | | | | |

Supp 3 continued

| **Study (date)** | **2-minute walk test**  **(m)** | | **6-minute walk test**  **(m)** | | **10-metre walk test**  **(m/s)** | | **Timed up-and-go test**  **(s)** | | **Rating of perceived exertion**  **(0-10)** | |
| --- | --- | --- | --- | --- | --- | --- | --- | --- | --- | --- |
|  | Pre-RAGT | Post-RAGT | Pre-RAGT | Post-RAGT | Pre-RAGT | Post-RAGT | Pre-RAGT | Post-RAGT | Pre-RAGT | Post-RAGT |
| Lam  et al. (2015) |  |  | Loko-R  100 ± 102  Control  120 ± 138 | Loko-R  122 ± 117  Control  149 ± 170  Within groups p>0.05 | Loko-R  0.29 ± 0.28  Control  0.33 ± 0.35 | Loko-R  0.40 ± 0.31  Control  0.44 ± 0.45  Within groups p>0.05 |  |  | Loko-R  4.0 ± 1.6  Control  2.5 ± 1.2 | Loko-R  4.1 ± 1.2  Control  2.6 ± 0.5  Time effect F(1,11)=  0.06 (p=0.81)  Interaction F(1,11)=  8.7  (p=0.01) |
| Okawara  et al. (2020) | Overall group  22 ± 22  High ability group  23 ± 22  Low ability group*  4.0 | Overall group  28 ± 22 (p=0.05;  95% CI -0.5-12)  High ability group  29 ± 22 (p=0.07;  95% CI -0.9-12)  Low ability group*  8.0 |  |  | High ability group  0.26 ± 0.27 | High ability  group  0.34 ± 0.30 (p=0.01;  95% CI 0.02-0.14)  Low ability  Group^†^ | Overall group  92 ± 73  High ability group  84 ± 77  Low ability group*  103 | Overall group  75 ± 63  (p<0.01;  95% CI -7.6, -26)  High ability  group  69 ± 66  (p=0.01;  95% CI -6.5, -26)  Low ability  group*  83 | Overall group^¶^ 12.6±1.7 | Overall group**^¶^** 11.9 ± 1.3 (p=0.03; 95% CI 0.0, -1.7) |
| Only outcomes that were assessed in more than one of the included studies have been presented.  Outcome data presented as mean ± SD where this information was provided in the source article. Statistical data presented where this information was provided in the source article.  *Only 1 participant completed the test in the Low Ability Group; ^†^No participants completed the test in the Low Ability Group; **^¶^** RPE measured on a 6-20 scale; CI = Confidence Interval | | | | | | | | | | |

Supp 3 continued

|  | **2-minute walk test**  **(m)** | | **6-minute walk test**  **(m)** | | **10-metre walk test**  **(m/s)** | | **Timed up-and-go test**  **(s)** | | **Rating of perceived exertion**  **(0-10)** | |
| --- | --- | --- | --- | --- | --- | --- | --- | --- | --- | --- |
| **Study (date)** | Pre-RAGT | Post-RAGT | Pre-RAGT | Post-RAGT | Pre-RAGT | Post-RAGT | Pre-RAGT | Post-RAGT | Pre-RAGT | Post-RAGT |
| Piira  et al. (2019) |  |  | RAGT group  82.3  (range  25.0-214.5)  Control  170.4  (range  63.0-390.0) | RAGT group mean change  6.6  (range  -14.0-34.0; p=0.25)  Control mean change  23.1  (range  -45.0-43.0; p=0.59)  Between groups p=0.61,  r=-0.07 | RAGT group  0.3  (range 0.1-0.7)  Control  0.6  (range 0.1-1.0) | RAGT group  mean change  0  (range -0.1-0.1; p=0.80)  Control mean change  0.1  (range -0.1-0.6; p=0.44)  Between groups p=0.61, r=-0.15 |  |  |  |  |
| Rodriguez-Fernandez  et al. (2025)* |  |  | RAGT group  35.5 ± 28.2  Control  38.9 ± 28.9  Between groups  p=0.42 | RAGT group  47.1 ± 29.9  Control  46.3 ± 30.8  Time effect  p<0.05  Between groups  p=1.0 | RAGT group  0.14 ± 0.11  Control  0.14 ± 0.09  Between groups  p=1.0 | RAGT group  0.17 ± 0.10  Control  0.14 ± 0.11  Time effect^⁋^  p=0.009  Between  groups  p=0.69 | RAGT group  123.9 ± 53.7  Control 125.8 ± 92.8  Between groups  p=0.84 | RAGT group  102.7 ± 52.7  Control  101.2 ± 71.8  Time effect^⁋^  p=0.006  Between groups  p=0.55 |  |  |
| Only outcomes that were assessed in more than one of the included studies have been presented.  Outcome data presented as mean ± SD where this information was provided in the source article. Statistical data presented where this information was provided in the source article.  *outcomes recorded during 5^th^ and 10^th^ (final) RAGT sessions; ^⁋^for Control group only (RAGT group p-value not reported). | | | | | | | | | | |

Supp 3 continued

|  | **2-minute walk test**  **(m)** | | **6-minute walk test**  **(m)** | | **10-metre walk test**  **(m/s)** | | **Timed up-and-go test**  **(s)** | | **Rating of perceived exertion**  **(0-10)** | |
| --- | --- | --- | --- | --- | --- | --- | --- | --- | --- | --- |
| **Study (date)** | Pre-RAGT | Post-RAGT | Pre-RAGT | Post-RAGT | Pre-RAGT | Post-RAGT | Pre-RAGT | Post-RAGT | Pre-RAGT | Post-RAGT |
| Stampacchia  et al. (2020)^⁋^ |  |  | Group A  124.1 ± 50.9  Group B  36.2 ± 10.9 | Group A  196.3 ± 92.6 (p=0.01)  Group B  49.0 ± 10.7 (p<0.001) | Group A  0.38 ± 0.08  Group B  0.10 ± 0.03 | Group A  0.51 ± 0.07 (p=0.009)  Group B  0.13 ± 0.04 (p<0.001) | Group A  31.4 ± 17.9  Group B  99.0 ± 44.2 | Group A  21.9 ± 11.4 (p=0.045)  Group B  69.0 ± 20.0 (p<0.001) |  |  |
| Tsai et al. (2024) |  |  |  |  |  |  |  |  | Not reported | RAGT group  14 ± 2**^¶^** |
| Williams  et al. (2021) |  |  |  |  | EKSO  1.05 ± 0.16 | EKSO  1.43 ± 0.4 |  |  | EKSO  3.3 ± 1.2  Lokomat  0.5 ± 1.1 | EKSO  4.0 ± 1.9  Lokomat  1.4 ± 1.3 |
| Only outcomes that were assessed in more than one of the included studies have been presented.  Outcome data presented as mean ± SD where this information was provided in the source article. Statistical data presented where this information was provided in the source article.  ^⁋^Group A performed the tests without the exoskeleton and Group B performed the tests with the exoskeleton; **^¶^** RPE measured on a 6-20 scale. | | | | | | | | | | |
