# Supplementary material for: The effect of robot-assisted gait training on physical activity outcomes in people with spinal cord injury: A systematic review
Source: Clin Rehabil. 2026 Feb 18;40(6):734–56. doi: 10.1177/02692155251411864 (PMC13191083; doi:10.1177/02692155251411864)
Supplement: sj-docx-4-cre-10.1177_02692155251411864 - Supplemental material for The effect of robot-assisted gait training on physical activity outcomes in people with spinal cord injury: A systematic review [file sj-docx-4-cre-10.1177_02692155251411864.docx]

Supp 4: Summary of certainty of evidence assessment for change in functional outcomes before and after RAGT

| **Outcome** | **Effect over time** | **Number of participants**  **(study design type)** | **Certainty of evidence**^‡^  **(GRADE assessment)** |
| --- | --- | --- | --- |
| 2-minute walk test | 2/2 studies found a pre- to post-RAGT increase in distance. One indicated that this difference was significant (p=0.05), while the other only provided descriptive statistics. | 23  (1 NRNC and 1 case series) | Very low ⊕OOO  (due to serious study limitations, indirectness, and imprecision) |
| 6-minute walk test | 9/9 studies found a pre- to post-RAGT increase in distance. Six indicated that this difference was significant (p<0.05); the others indicated that the difference was not significant (p>0.05). | 287  (4 randomised*;  1 NRC; 4 NRNC*) | Very low ⊕OOO  (due to borderline indirectness and serious study limitations and imprecision) |
| 10-metre walk test | 12/13 studies found a pre- to post-RAGT increase in speed, while one found no change. Six indicated that this difference was significant (p<0.01), three indicated that this difference was not significant (p>0.05), and four only provided descriptive statistics. | 330  (5 randomised^†^; 1 NRC;  6 NRNC*; 1 case series) | Very low ⊕OOO  (due to borderline inconsistency and serious study limitations, indirectness, and imprecision) |
| Timed  up-and-go test | 7/7 studies found a pre- to post-RAGT decrease in time. Five indicated that this difference was significant (p<0.05), while the remaining two studies only reported descriptive statistics. | 196  (2 randomised;  1 NRC; 4 NRNC*) | Very low ⊕OOO  (due to serious study limitations, indirectness, and imprecision) |
| Rating of perceived exertion | 2/3 studies found a pre- to post-RAGT increase, while one found a decrease. The latter study indicated that the decrease was significant (p<0.05), while one of the former studies indicated that the observed increase was not significant (p>0.05). The remaining study only reported descriptive statistics. | 43  (2 randomised^†^ and 1 NRNC) | Very low ⊕OOO  (due to serious study limitations, indirectness, imprecision, and inconsistency) |
| *including 1 pilot study; ^†^including 2 pilot studies; ^‡^see Table 11 for details. GRADE = Grading of Recommendation, Assessment, Development and Evaluation;  NRC = Non-randomised comparative; NRNC = Non-randomised non-comparative; RAGT = Robot-Assisted Gait Training | | | |
