# Supplementary material for: The effect of robot-assisted gait training on physical activity outcomes in people with spinal cord injury: A systematic review
Source: Clin Rehabil. 2026 Feb 18;40(6):734–56. doi: 10.1177/02692155251411864 (PMC13191083; doi:10.1177/02692155251411864)
Supplement: sj-docx-5-cre-10.1177_02692155251411864 - Supplemental material for The effect of robot-assisted gait training on physical activity outcomes in people with spinal cord injury: A systematic review [file sj-docx-5-cre-10.1177_02692155251411864.docx]

Supp 5: Justification for certainty of evidence ratings of physical activity outcome changes across the Robot-Assisted Gait Training (RAGT) period.

| **Outcome** | **GRADE domain** | **Judgement** | **Level of concern** |
| --- | --- | --- | --- |
| Up time (min) | Study limitations | *Randomised trial:* The single randomised trial that reported up time (10 participants) did not conceal participant allocation or blind subjects, therapists, or assessors (Rodríguez-Fernández et al., 2025).  *Non-randomised trials:* All 10 studies had a high RoB for the effect of confounders. Additionally, 7 studies (1, 2, 7, 3, 1, 59, and 24 participants) had a high RoB for lack of blinding of assessors (Chang et al., 2020; Hotz et al., 2024; Kolakowsky-Hayner, 2013; Kressler et al., 2014; Lester & Gorgey, 2018; Swank et al., 2020; Wright et al., 2023). Three studies (13, 99 and 2 participants) had high RoB related to comparability of the target group (Gagnon et al., 2018; Gillespie et al., 2023; Hotz et al., 2024). Five studies (13, 99, 2, 1, and 59 participants) had high RoB for target group selection (Gagnon et al., 2018; Gillespie et al., 2023; Hotz et al., 2024; Lester & Gorgey, 2018; Swank et al., 2020). Therefore, we judged the trials to have serious methodological limitations. | Serious |
|  | Indirectness | The patients and interventions in all 11 studies provided direct evidence of the observed improvement over time. There was one randomised trial within which the control group received “standard care” (Rodríguez-Fernández et al., 2025). Six studies were non-comparative, while the control group in the one comparative study did not undergo an alternative form of gait training meaning that a comparison of “Up time” was not possible (Faulkner et al., 2021). Overall, there was very limited direct comparison between RAGT and other forms of gait training, which was considered a serious factor for indirectness. | Serious |
|  | Imprecision | The total number of patients in the relevant trials was 231. Two studies (22 participants) presented results of statistical analyses (Faulkner et al., 2021; Rodríguez-Fernández et al., 2025), though one was a pilot study and confidence intervals were not reported. The remaining studies only presented descriptive statistics. Therefore, we judged the trials to have serious imprecision. | Serious |
|  | Inconsistency | All 11 studies reported an increase in “Up time” from the beginning to the end of RAGT. | Not serious |
|  | Publication bias | Although the relevant studies comprised solely positive findings from small-scale trials, we did not suspect publication bias due to the lack of industry-involvement and conflicts of interest reported within them, and the comprehensive nature of the systematic review search. | Not suspected |
| RAGT = Robot-Assisted Gait Training; RoB = Risk of Bias | | | |

Supp 5 continued

| **Outcome** | **GRADE domain** | **Judgement** | **Level of concern** |
| --- | --- | --- | --- |
| Walk time (mins) | Study limitations | *Randomised trial:* The single randomised trial that reported up time (10 participants) did not conceal participant allocation or blind subjects, therapists, or assessors (Rodríguez-Fernández et al., 2025).  *Non-randomised trials:* All 14 studies had a high RoB for the effect of confounders. Additionally, 9 studies (1, 55, 1, 7, 3, 1, 20, 59, and 24 participants) had a high RoB for lack of blinding assessors (Chang et al., 2020; Grasmücke et al., 2017; Ikumi et al., 2017; Kolakowsky-Hayner, 2013; Kressler et al., 2014; Lester & Gorgey, 2018; Okawara et al., 2020; Swank et al., 2020; Wright et al., 2023). 5 studies (10, 13, 99, 55, and 20 participants) had a high RoB related to comparability to the target group (Fleerkotte et al., 2014; Gagnon et al., 2018; Gillespie et al., 2023; Grasmücke et al., 2017; Okawara et al., 2020). 4 studies (13, 99, 1, and 59 participants) had a high RoB related to the target group selection (Gagnon et al., 2018; Gillespie et al., 2023; Lester & Gorgey, 2018; Swank et al., 2020). Therefore, we judged the trials to have serious methodological limitations. | Serious |
|  | Indirectness | The patients and interventions in all 15 studies provided direct evidence of an observed improvement over time. There was one randomised trial within which the control group received “standard care” (Rodríguez-Fernández et al., 2025). 13/15 studies were non-comparative, while the control group in the one comparative study did not undergo an alternative form of gait training meaning that a comparison of “Walk time” was not possible (Faulkner et al., 2021). As such, there was very limited direct comparison between RAGT and other forms of gait training, which was considered a serious factor for indirectness. | Serious |
|  | Imprecision | The total number of patients in the relevant trials was 365. Six studies (171 participants) presented data from statistical analyses and indicated that observed improvements were significant, though confidence intervals were not reported. The remaining studies only presented descriptive statistics. Therefore, we judged the trials to have serious imprecision. | Serious |
|  | Inconsistency | All 15 studies reported an increase in “Up time” from the beginning to the end of RAGT. | Not serious |
|  | Publication bias | Although the relevant studies contained no negative findings and comprised of several small-scale trials, we did not suspect publication bias due to the lack of industry-involvement and conflicts of interest reported within them, and the comprehensive nature of the systematic review search. | Not suspected |
| RAGT = Robot-Assisted Gait Training; RoB = Risk of Bias | | | |

Supp 5 continued

| **Outcome** | **GRADE domain** | **Judgement** | **Level of concern** |
| --- | --- | --- | --- |
| Walk speed (m/s) | Study limitations | *Randomised trials*: all 5 were limited for reasons related to a lack of blinding or allocation concealment. 1 study (8 participants) had heterogenous groups at baseline and only obtained outcome data for 80% of subjects (Wu et al., 2012). 1 study (5 participants) did not report statistical analysis data within or between groups (Williams et al., 2021).  *Non-randomised trials*: all 8 non-randomised trials had a high RoB for the effect of confounders. Five studies (10, 55, 2, 20 and 25 participants) had a high RoB related to comparability to the target group (Fleerkotte et al., 2014; Grasmücke et al., 2017; Hotz et al., 2024; Okawara et al., 2020; Stampacchia et al., 2020). Five studies (55, 2, 11, 20 and 24 participants) had high RoB for lack of blinding assessors (Grasmücke et al., 2017; Hotz et al., 2024; Khan et al., 2019; Okawara et al., 2020; Wright et al., 2023). Two studies (2, 11 participants) had high RoB related to the target group selection (Hotz et al., 2024; Khan et al., 2019). | Serious |
|  | Indirectness | The patients and interventions in all 13 studies provided direct evidence of an observed improvement over time. All 8 non-randomised studies were non-comparative, while the control groups in the 5 randomised trials all underwent unique gait training protocols, only one of which consisted of standard gait training without an exoskeleton (Rodríguez-Fernández et al., 2025). Therefore, there was a serious risk of indirectness. | Serious |
|  | Imprecision | The total number of patients across the relevant trials was 248. Nine studies (233 participants) presented data from statistical analyses and all but one indicated that observed improvements were significant, though confidence intervals were not reported. The remaining studies only presented descriptive statistics. Therefore, we judged the trials to have serious imprecision. | Serious |
|  | Inconsistency | All 13 studies indicated an improvement in walk speed across the RAGT period. | Not serious |
|  | Publication bias | Although the relevant studies contained no negative findings and comprised of several small-scale trials, we did not suspect publication bias due to the limited apparent industry-involvement and conflicts of interest reported within them, and the comprehensive nature of the systematic review search. | Not suspected |
| RAGT = Robot-Assisted Gait Training; RoB = Risk of Bias | | | |

Supp 5 continued

| **Outcome** | **GRADE domain** | **Judgement** | **Level of concern** |
| --- | --- | --- | --- |
| Walk distance (m) | Study limitations | *Randomised trials:* All 3 studies were limited for reasons related to a lack of blinding or allocation concealment. 1 study (8 participants) had heterogenous groups at baseline and only obtained outcome data for 80% of subjects (Wu et al., 2012). The other study (5 participants) did not report statistical analysis data within or between groups (Williams et al., 2021).  *Non-randomised trials:* all 9 studies had a high RoB for the effect of confounders. Eight studies (55, 2, 1, 11, 7, 3, 20 and 24 participants) had high RoB for lack of blinding assessors (Grasmücke et al., 2017; Hotz et al., 2024; Ikumi et al., 2017; Khan et al., 2019; Kolakowsky-Hayner, 2013; Kressler et al., 2014; Okawara et al., 2020; Wright et al., 2023). Three studies (55, 2 and 20 participants) had a high RoB related to comparability to the target group (Grasmücke et al., 2017; Hotz et al., 2024; Okawara et al., 2020). Two studies (2, 11 participants) had high RoB for target group selection (Hotz et al., 2024; Khan et al., 2019). | Serious |
|  | Indirectness | The patients and interventions in all 12 studies provided direct evidence of an observed improvement over time. All 9 non-randomised studies were non-comparative, while the control groups in the 3 randomised trials underwent unique gait training protocols, only one of which consisted of standard gait training without an exoskeleton (Rodríguez-Fernández et al., 2025). Therefore, there was a serious risk of indirectness. | Serious |
|  | Imprecision | The total number of participants across the relevant studies was 193. Five studies (159 participants) presented data from statistical analyses and indicated that observed improvements were significant, though confidence intervals were not reported. The remaining studies only presented descriptive statistics. Therefore, we judged the trials to have serious imprecision. | Serious |
|  | Inconsistency | All 12 studies indicated an improvement in walk distance across the RAGT period. | Not serious |
|  | Publication bias | Although the relevant studies contained no negative findings and comprised of several small-scale trials, we did not suspect publication bias due to the limited apparent industry-involvement and conflicts of interest reported within them, and the comprehensive nature of the systematic review search. | Not suspected |
| RAGT = Robot-Assisted Gait Training; RoB = Risk of Bias | | | |

Supp 5 continued

| **Outcome** | **GRADE domain** | **Judgement** | **Level of concern** |
| --- | --- | --- | --- |
| Steps (n) | Study limitations | *Randomised trials:* All 3 studies were limited for reasons related to a lack of blinding or concealment. 1 study (50 participants) had heterogenous groups at baseline (Hong et al., 2020). Another study (5 participants) did not report statistical analysis data within or between groups (Williams et al., 2021).  *Non-randomised trials:* all 11 studies had a high RoB for the effect of confounders. Seven studies (1, 2, 11, 3, 1, 59 and 8 participants) had high RoB for lack of blinding assessors (Chang et al., 2020; Hotz et al., 2024; Khan et al., 2019; Kressler et al., 2014; Lester & Gorgey, 2018; Swank et al., 2020; Wright et al., 2023). Six studies (13, 99, 2, 11, 1 and 59 participants) had high RoB for target group selection (Gagnon et al., 2018; Gillespie et al., 2023; Hotz et al., 2024; Khan et al., 2019; Lester & Gorgey, 2018; Swank et al., 2020). Three studies (13, 99 and 2 participants) had a high RoB related to comparability to the target group (Gagnon et al., 2018; Gillespie et al., 2023; Hotz et al., 2024). | Serious |
|  | Indirectness | The patients and interventions in 13/14 studies provided direct evidence of an observed improvement over time, while one study provided direct evidence of an observed decrease over time (Aach et al., 2023). 10/11 non-randomised trials were non-comparative, while the control group in the one comparative study did not undergo an alternative form of gait training meaning that a comparison of “Walk time” was not possible (Faulkner et al., 2021). The control groups in the 3 randomised trials underwent unique gait training protocols, only one of which consisted of standard gait training without an exoskeleton (Rodríguez-Fernández et al., 2025). Therefore, there was a serious risk of indirectness. | Serious |
|  | Imprecision | The total number of participants across the relevant studies was 338. Three studies (46 participants) indicated that observed improvements were statistically significant, though confidence intervals were not reported. The one study that observed a decrease over time (50 participants) indicated that the difference was statistically significant (Aach et al., 2023). The remaining studies only presented descriptive statistics. Therefore, we judged the trials to have serious imprecision. | Serious |
|  | Inconsistency | 13/14 studies (288 participants) indicated an improvement in steps across the RAGT period, while the remaining study (50 participants) indicated a decrease. Therefore, we judged the trials to have borderline inconsistency. | Borderline |
|  | Publication bias | Although the relevant studies contained no negative findings and comprised of several small-scale trials, we did not suspect publication bias due to the limited apparent industry-involvement and conflicts of interest reported within them, and the comprehensive nature of the systematic review search. | Not suspected |
| RAGT = Robot-Assisted Gait Training; RoB = Risk of Bias | | | |
