# Supplementary material for: The effect of robot-assisted gait training on physical activity outcomes in people with spinal cord injury: A systematic review
Source: Clin Rehabil. 2026 Feb 18;40(6):734–56. doi: 10.1177/02692155251411864 (PMC13191083; doi:10.1177/02692155251411864)
Supplement: sj-docx-6-cre-10.1177_02692155251411864 - Supplemental material for The effect of robot-assisted gait training on physical activity outcomes in people with spinal cord injury: A systematic review [file sj-docx-6-cre-10.1177_02692155251411864.docx]

Supp 6: Justification for certainty of evidence ratings of functional outcome changes across the Robot-Assisted Gait Training (RAGT) period.

| **Outcome** | **GRADE domain** | **Judgement** | **Level of concern** |
| --- | --- | --- | --- |
| 2-minute walk test | Study limitations | Both studies had a high RoB for the effect of confounders and lack of blinding. Additionally, 1 study had a high RoB related to the comparability of the target group (Okawara et al., 2020). Therefore, we judged the trials to have serious methodological limitations. | Serious |
|  | Indirectness | The patients and interventions in both studies provided direct evidence of the observed improvement over time. Both studies were non-comparative. As such, there was no direct comparison between RAGT and other forms of gait training, which was considered a serious factor for indirectness. | Serious |
|  | Imprecision | The total number of patients in the relevant trials was 23. 1 study (20 participants) presented results of statistical analyses (Okawara et al., 2020), which included a wide 95% confidence interval for the overall group (-0.5-12 m) relative to the mean difference (6 m). The remaining study only presented descriptive statistics (Kressler et al., 2014). Therefore, we judged the trials to have serious imprecision. | Serious |
|  | Inconsistency | Both studies reported an increase in the 2-minute walk test from the beginning to the end of the RAGT period. | Not serious |
|  | Publication bias | Although the relevant studies comprised solely positive findings from small-scale trials, we did not suspect publication bias due to the lack of industry-involvement and conflicts of interest reported within them, and the comprehensive nature of the systematic review search. | Not suspected |
| RAGT = Robot-Assisted Gait Training; RoB = Risk of Bias | | | |

Supp 6 continued

| **Outcome** | **GRADE domain** | **Judgement** | **Level of concern** |
| --- | --- | --- | --- |
| 6-minute walk test | Study limitations | *Randomised trials:* All 4 studies were limited for reasons related to lack of blinding or allocation concealment. 2 studies (50 and 24 participants) had heterogenous groups at baseline (Hong et al., 2020; Piira et al., 2019). 1 study (24 participants) was only able to collect complete data for 79% of participants and did not conduct analysis by “intention to treat” (Piira et al., 2019).  *Non-randomised trials:* All 5 studies had a high RoB for the effect of confounders. Additionally, 4 studies (12, 70, 2 and 52 participants) had high RoB related to comparability of the target group (Fleerkotte et al., 2014; Grasmücke et al., 2017; Hotz et al., 2024; Stampacchia et al., 2020), and 2 studies (70 and 2 participants) had high RoB for lack of blinding assessors (Grasmücke et al., 2017; Hotz et al., 2024). One study have high RoB for target group selection (Hotz et al., 2024). Therefore, we judged the trials to have serious methodological limitations. | Serious |
|  | Indirectness | The patients and interventions in all 9 studies provided direct evidence of an observed improvement over time. 3/5 comparative studies also provided direct evidence of an observed improvement over time (Hong et al., 2020; Piira et al., 2019; Rodríguez-Fernández et al., 2025). The control groups in the remaining comparative studies underwent unique gait training protocols, neither of which consisted of standard training without an exoskeleton (Lam et al., 2015; Stampacchia et al., 2020). 4 studies were non-comparative. Overall, there was a borderline risk of indirectness. | Borderline |
|  | Imprecision | The total number of patients in the relevant trials was 287. 8/9 studies presented data from statistical analyses but only 6 (244 participants) found improvements to be significant, while 2 studies (41 participants) found the improvements to be non-significant, and the final study did not present statistical data (Hotz et al., 2024). None of the studies reported confidence intervals. Therefore, we judged the trials to have serious imprecision. | Serious |
|  | Inconsistency | The direction of the effect was in favour of a substantial improvement over time in all 9 studies. | Not serious |
|  | Publication bias | Although the relevant studies contained no negative findings and comprised of several small-scale trials, we did not suspect publication bias due to the lack of industry-involvement and conflicts of interest reported within them, and the comprehensive nature of the review search. | Not suspected |
| RoB = Risk of Bias | | | |

Supp 6 continued

| **Outcome** | **GRADE domain** | **Judgement** | **Level of concern** |
| --- | --- | --- | --- |
| 10-metre walk test | Study limitations | *Randomised trials*: all 5 were limited for reasons related to a lack of blinding and allocation concealment. 2 studies (24 and 50 participants) had heterogenous groups at baseline (Hong et al., 2020; Piira et al., 2019). 2 studies (6 and 24 participants) only obtained outcome data for <85% of subjects (Piira et al., 2019; Williams et al., 2021). 1 study (24 participants) did not analyse data by “intentio n to treat” (Piira et al., 2019). 1 study (6 participants) did not report statistical analysis data within or between groups (Williams et al., 2021).  *Non-randomised trials*: all 8 non-randomised trials had a high RoB for the effect of confounders. Six studies (12, 14, 20, 2, 52 and 70 participants) had a high RoB related to comparability to the target group (Fleerkotte et al., 2014; Gagnon et al., 2018; Grasmücke et al., 2017; Hotz et al., 2024; Okawara et al., 2020; Stampacchia et al., 2020). 3 studies (3, 2, 20 and 70 participants) had high RoB for lack of blinding assessors (Grasmücke et al., 2017; Hotz et al., 2024; Kressler et al., 2014; Okawara et al., 2020). Two studies (14 and 2 participants) had high RoB related to the target group selection (Gagnon et al., 2018; Hotz et al., 2024). Therefore, we judged the trials to have serious methodological limitations. | Serious |
|  | Indirectness | The patients and interventions in 12/13 studies provided direct evidence of an observed improvement over time, though one study observed no change. The results of 3/5 randomised studies also provided direct evidence of an observed improvement over time (Hong et al., 2020; Piira et al., 2019; Rodríguez-Fernández et al., 2025). The control groups in the remaining randomised trials and the 1 non-randomised comparative trial all underwent unique gait training protocols, none of which consisted of standard training without an exoskeleton (Lam et al., 2015; Stampacchia et al., 2020; Williams et al., 2021). The remaining studies were non-comparative. Therefore, there was a serious risk of indirectness. | Serious |
|  | Imprecision | The total number of patients across the relevant trials was 330. Nine studies presented data from statistical analyses but only six (256 participants) indicated that observed improvements were significant, while three found the improvements to be non-significant (53 participants). A confidence interval was only reported for one sub-group (12 participants) in a single study (Okawara et al., 2020). The remaining studies only presented descriptive statistics. Therefore, we judged the trials to have serious imprecision. | Serious |
|  | Inconsistency | The direction of the effect was in favour of an improvement over time in 12/13 studies, but only six (including 1 RCT; 50 participants) reported this difference to be statistically significant. The remaining study was an RCT (24 participants) that reported no change over time. | Borderline |
|  | Publication bias | Although the relevant studies contained no negative findings and comprised of several small-scale trials, we did not suspect publication bias due to the limited apparent industry-involvement and conflicts of interest reported within them, and the comprehensive nature of the systematic review search. | Not suspected |
| RCT = Randomised Controlled Trial; RoB = Risk of Bias | | | |

Supp 6 continued

| **Outcome** | **GRADE domain** | **Judgement** | **Level of concern** |
| --- | --- | --- | --- |
| Timed  up-and-go test | Study limitations | *Randomised trials:* both studies were limited for reasons related to a lack of blinding or allocation concealment, while one study (50 participants) additionally had heterogenous groups at baseline (Hong et al., 2020).  *Non-randomised trials:* all 5 studies had a high RoB for the effect of confounders. Three studies (12, 2, 20 and 52 participants) had high RoB related to comparability of the target group (Fleerkotte et al., 2014; Hotz et al., 2024; Okawara et al., 2020; Stampacchia et al., 2020). Two studies (2 and 20 participants) had high RoB for lack of blinding assessors (Hotz et al., 2024; Okawara et al., 2020). One study (2 participants) also had high RoB for target group selection (Hotz et al., 2024). | Serious |
|  | Indirectness | The patients and interventions in all 7 studies provided direct evidence of an observed improvement over time. The results of the two randomised trials also provided direct evidence of an observed improvement over time (Hong et al., 2020; Rodríguez-Fernández et al., 2025). The control group in the one non-randomised comparative study did not undergo gait training without robotic assistance (Stampacchia et al., 2020). The remaining studies were non-comparative. Therefore, there was a serious risk of indirectness. | Serious |
|  | Imprecision | The total number of participants across the relevant studies was 196. Five studies presented data from statistical analyses and indicated that observed improvements were significant. Only one of these presented a confidence interval (Okawara et al., 2020), which was relatively wide (-7.6 to-26 s) compared to the mean difference (-17 s). The remaining studies did not provide statistical data for time effects. Overall, we judged the trials to have serious risk of imprecision. | Serious |
|  | Inconsistency | All 7 studies indicated an improvement in the TUG test from the beginning to the end of the RAGT period. | Not serious |
|  | Publication bias | Although the relevant studies contained no negative findings and comprised of several small-scale trials, we did not suspect publication bias due to the limited apparent industry-involvement and conflicts of interest reported within them, and the comprehensive nature of the systematic review search. | Not suspected |
| RAGT = Robot-Assisted Gait Training; RoB = Risk of Bias; TUG = Timed Up-and-Go | | | |

Supp 6 continued

| **Outcome** | **GRADE domain** | **Judgement** | **Level of concern** |
| --- | --- | --- | --- |
| Rating of Perceived Exertion | Study limitations | *Randomised trials:* Both randomised studies (6 and 17 participants) were limited for reasons related to lack of blinding (Lam et al., 2015; Williams et al., 2021). One study (6 participants) only obtained data for 83% of participants and did not report statistical analysis data within or between groups (Williams et al., 2021).  *Non-randomised trial:* the single relevant study (20 participants) had a high RoB for the effect of confounders, comparability of the target group, and lack of blinding of assessors (Okawara et al., 2020). | Serious |
|  | Indirectness | The patients and interventions in all 3 studies provided direct evidence for their observed changes over time. The control groups of the 2 randomised studies underwent unique gait training protocols, neither of which consisted of standard training without an exoskeleton. The remaining study was non-comparative. | Serious |
|  | Imprecision | The total number of participants across the relevant studies was 43. One randomised study observed a statistically non-significant increase over time (Lam et al., 2015), while the single non-randomised study observed a statistically significant decrease (Okawara et al., 2020). The latter study presented a wide confidence interval (0.0 to -1.7) relative to the mean difference (-0.7). The remaining randomised study only reported descriptive statistics. Therefore, we judged there to be a serious risk of imprecision. | Serious |
|  | Inconsistency | 2/3 studies found an increase over time, though neither was reported as statistically significant. The remaining study found a statistically significant decrease over time. | Serious |
|  | Publication bias | Although the relevant studies contained no negative findings and comprised of several small-scale trials, we did not suspect publication bias due to the limited apparent industry-involvement and conflicts of interest reported within them, and the comprehensive nature of the systematic review search. | Not suspected |
| RoB = Risk of Bias | | | |
